# The effects of aerobic exercise on neuroimmune responses in animals with traumatic peripheral nerve injury: a systematic review with meta-analyses

**DOI:** 10.1186/s12974-023-02777-y

**Published:** 2023-05-03

**Authors:** Marije L. S. Sleijser-Koehorst, Meghan A. Koop, Michel W. Coppieters, Ivo J. Lutke Schipholt, Nemanja Radisic, Carlijn R. Hooijmans, Gwendolyne G. M. Scholten-Peeters

**Affiliations:** 1 Department of Human Movement Sciences, Faculty of Behavioural and Movement Sciences, Amsterdam Movement Sciences-Program Musculoskeletal Health, Vrije Universiteit Amsterdam, Van Der Boechorststraat 9, 1081 BT Amsterdam, The Netherlands; 2 Menzies Health Institute Queensland, Griffith University, Brisbane and Gold Coast, Australia; 3 School of Health Sciences and Social Work, Griffith University, Brisbane and Gold Coast, Australia; 4 Department of Clinical Chemistry, Laboratory Medical Immunology, Amsterdam University Medical Centre, Location VUmc, Amsterdam, The Netherlands; 5 Department of Anesthesiology, Pain and Palliative Care (Meta Research Team), Radboud University Medical Centre, Nijmegen, The Netherlands

**Keywords:** Peripheral neuropathy, Mechanisms, Exercise, Physical activity, Neuro-immune system, Cytokines, Neurotrophins, Macrophages, Neuroinflammation, Neurotransmitters

## Abstract

**Background:**

Increasing pre-clinical evidence suggests that aerobic exercise positively modulates neuroimmune responses following traumatic nerve injury. However, meta-analyses on neuroimmune outcomes are currently still lacking. This study aimed to synthesize the pre-clinical literature on the effects of aerobic exercise on neuroimmune responses following peripheral nerve injury.

**Methods:**

MEDLINE (via Pubmed), EMBASE and Web of Science were searched. Controlled experimental studies on the effect of aerobic exercise on neuroimmune responses in animals with a traumatically induced peripheral neuropathy were considered. Study selection, risk of bias assessment and data extraction were performed independently by two reviewers. Results were analyzed using random effects models and reported as standardized mean differences. Outcome measures were reported per anatomical location and per class of neuro-immune substance.

**Results:**

The literature search resulted in 14,590 records. Forty studies were included, reporting 139 comparisons of neuroimmune responses at various anatomical locations. All studies had an unclear risk of bias. Compared to non-exercised animals, meta-analyses showed the following main differences in exercised animals: (1) in the affected nerve, tumor necrosis factor-α (TNF-α) levels were lower (*p* = 0.003), while insulin-like growth factor-1 (IGF-1) (*p* < 0.001) and Growth Associated Protein 43 (GAP43) (*p* = 0.01) levels were higher; (2) At the dorsal root ganglia, brain-derived neurotrophic factor (BDNF)/BDNF mRNA levels (*p* = 0.004) and nerve growth factor (NGF)/NGF mRNA (*p* < 0.05) levels were lower; (3) in the spinal cord, BDNF levels (*p* = 0.006) were lower; at the dorsal horn, microglia (*p* < 0.001) and astrocyte (*p* = 0.005) marker levels were lower; at the ventral horn, astrocyte marker levels (*p* < 0.001) were higher, and several outcomes related to synaptic stripping were favorably altered; (4) brainstem 5-HT2A receptor levels were higher (*p* = 0.001); (5) in muscles, BDNF levels (*p* < 0.001) were higher and TNF-α levels lower (*p* < 0.05); (6) no significant differences were found for systemic neuroimmune responses in blood or serum.

**Conclusion:**

This review revealed widespread positive modulatory effects of aerobic exercise on neuroimmune responses following traumatic peripheral nerve injury. These changes are in line with a beneficial influence on pro-inflammatory processes and increased anti-inflammatory responses. Given the small sample sizes and the unclear risk of bias of the studies, results should be interpreted with caution.

**Supplementary Information:**

The online version contains supplementary material available at 10.1186/s12974-023-02777-y.

## Background

Peripheral neuropathy is a common disorder in which the peripheral nervous system is affected [1, 2]. People with peripheral neuropathy often report numbness, paresthesia and/or muscle weakness [2]. Neuropathic pain is also a common symptom in people with peripheral neuropathy, and contributes to poor quality of life [3, 4]. Moreover, people with neuropathic pain more frequently experience severe pain, comorbidities, difficulties with work participation, insomnia, anxiety and depression compared to people with non-neuropathic chronic pain [5].

Exercise is an important part of chronic pain management in people with neuropathic pain as it positively influences pain, physical functioning, general well-being and quality of life [6–8]. To date, the body of knowledge on the beneficial effects of exercise for peripheral neuropathy in human studies mostly focuses on non-traumatic nerve injuries, such as chemotherapy-induced peripheral neuropathy [7]. The beneficial effects of exercise in traumatic peripheral neuropathies (e.g., nerve lesion, crush or constriction) have however been extensively studied in animals [6, 7, 9]. Pre-clinical reviews showed that exercise has a positive effect on neuropathic pain, axon regeneration, and functional recovery [6, 7, 9].

Over the past decades, the potential of aerobic exercise to positively influence neuroimmune processes that occur after peripheral nerve injury has gained increased attention [6]. Considering the invasive nature of the majority of methods used to determine neuroimmune responses in the nervous system, it is difficult and often impossible to assess these outcomes in humans, which is why these outcomes have been mainly studied in animals.

As a response to peripheral nerve injury, several neuroimmune changes occur at the site of the injury, the dorsal root ganglion (DRG), spinal cord and in higher brain areas [10, 11]. At these sites, immune cells, such as macrophages, mast cells and glial cells, are recruited and upregulated, and release mediators (e.g., neurotrophins, cytokines and reactive oxygen species) that lead to sensitization of the pain neuraxis [11, 12]. Consequently, anti-inflammatory and pro-resolving mediators are released, resulting in an active biochemical program that enables inflamed tissues to return to the pre-inflammatory state and to prevent chronic neuropathic pain [11, 13]. However, a prolonged and an exaggerated inflammatory response might lead to persistent sensitization within the neuraxis and predispose the transition from acute to chronic neuropathic pain [11, 13]. Regular exercise after nerve injury has been associated with neuroprotective effects and a general improvement in immune function, thereby preventing a prolonged and exaggerated inflammatory response [14].

A thorough review of the literature helps to gain insights into how aerobic exercise influences neuroimmune processes that occur after peripheral nerve injury. Furthermore, it is important to identify gaps in the current body of knowledge, and provide recommendations for future research. To date, no systematic review with meta-analyses with this focus has been performed. Therefore, the aim of this systematic review was to summarize the effects of aerobic exercise on neuroimmune responses in animals with a traumatic peripheral neuropathy.

## Methods

This review has been designed and is reported in line with the Preferred Reporting Items for Systematic reviews and Meta-Analyses (PRISMA) 2020 statement [15]. The protocol has been registered in the International prospective register of systematic reviews (PROSPERO; registration number CRD42021245911).

### Literature search

A literature search was developed and conducted with the assistance of a research librarian (see Additional file 1). The following medical databases were searched from inception up to June 2020: MEDLINE (via Pubmed), EMBASE and Web of Science. Additionally, references from included studies were checked for potentially eligible studies.

### Study selection

The study selection was performed independently by two researchers (MSK, MK) using Rayyan [16]. The title and abstract of all studies were checked for potential eligibility. Then, the full text of the potentially eligible studies was screened to determine whether they met predetermined selection criteria. Discrepancies in study selection were discussed among the two researchers. If consensus could not be reached, a third reviewer (GSP or ILS) was consulted.

Studies were eligible for inclusion if: (1) a controlled experimental animal study was conducted; (2) animals with a traumatically induced peripheral neuropathy (e.g., nerve lesion, crush or constriction) were used; (3) intervention group(s) consisted of any form of aerobic exercise (e.g., treadmill running or swimming); (4) control group(s) consisted of animals with a traumatically induced peripheral neuropathy which did not receive any form of treatment (i.e., non-exercised animals); and (5) at least one neuroimmune response (i.e., processes or substances involved in interactions between the immune system and nervous system) was quantified. Criteria for exclusion were: (1) infant animals, pregnant animals, non-injured animals or animals with a systemic, auto-immune or neurological disease; (2) neuropathies acquired by illness or toxins (e.g., diabetic neuropathy, chemotherapy-induced peripheral neuropathy), hereditary neuropathies (e.g., Charcot–Marie–Tooth disease), cranial nerve neuropathy, experimental neuroma models, or laryngeal neuropathy; (3) multimodal treatment (i.e., aerobic exercise combined with other treatment interventions), passive exercise therapy (e.g., stretching, neuromobilizations), electrical stimulation, vehicle injections or sham graft injections near the site of injury. No studies were excluded based on language or publication date.

### Risk of bias assessment

Risk of bias assessment was performed independently by two researchers from a pool of three researchers (MSK, PT, NR). The risk of bias tool for animal studies developed by the Systematic Review Center for Laboratory Animal Experimentation (SYRCLE) was used [17]. This tool consists of 10 items that assess selection bias, performance bias, detection bias, attrition bias, reporting bias and other forms of bias. Items can be rated as ‘yes’, ‘no’ or ‘unclear’, indicating a high, low or unclear risk of bias, respectively. Differences in risk of bias scores were discussed among the two researchers. If necessary, a third researcher was consulted (MSK, PT, NR). The percentage agreement between the two researchers was calculated.

### Data extraction

Data were extracted independently by two researchers from a pool of three researchers (MSK, PT, NR), using a predetermined form. Information was gathered regarding study design, animals (e.g., species, strain, age), disease model (e.g., disease model used, location), intervention (e.g., type, duration, frequency, intensity), control group, relevant subgroups, outcome measures and results. When results were available for both the ipsilateral and contralateral side, we only extracted data for the experimental side (i.e., ipsilateral to the side of the lesion for the nervous system caudal and distal to the decussation, and contralateral to the lesion side for the nervous system cranial to the decussation). In case of discrepancies, the extracted information was discussed by the two researchers. If no consensus was reached, a third researcher was consulted (MSK, PT, NR). The authors of the papers were contacted if data were unclear or not reported in the article. If authors did not respond after a reminder, a universal desktop ruler (Universal Digitizer 3.8, AVP Soft) was used to extract data from figures by two researchers independently (MSK, NR).

### Data analyses

Data were analyzed using Review Manager (RevMan; Version 5.4, The Cochrane Collaboration, 2020). Differences in neuroimmune responses between experimental group(s) and control group(s) were expressed as standardized mean differences (SMDs) and 95% confidence intervals (95%CI), using a random effects model. For each meta-analysis, the number of studies (*N* = …) and number of comparisons (cf = …) are reported (e.g., *N* = 3; cf = 10)).

Outcome measures were organized per class of neuroimmune substance (e.g., neurotrophins, cytokines and neuroinflammation markers), and were reported per anatomical location (e.g., nerve, dorsal root ganglion, spinal cord). Meta-analyses were performed when at least two comparisons were available from a minimum of two original studies within the same anatomical location. Statistical heterogeneity was assessed using *I*^2^.

When a range of animals (e.g., 6–9) was reported rather than the exact number, the median of the range was used in the analyses. The control group size was corrected if a study compared multiple intervention groups to one control group (i.e., the number of control group animals was divided by the number of intervention groups, with minimally *N* = 2 per group). When a study measured the same outcomes repeatedly in the same anatomical location (e.g., in two different muscles, or multiple laminae), the largest SMD was retained [18, 19]. If at least 10 independent comparisons were available, formal subgroup analyses were conducted on type of animal, neuropathy, exercise or outcome. When sufficient low risk of bias studies were available, sensitivity analyses were performed to compare low risk of bias studies with all included studies. Publication bias was assessed by visual inspection of a funnel plot if at least 10 studies were available.

## Results

### Study selection

**Fig. 1:**
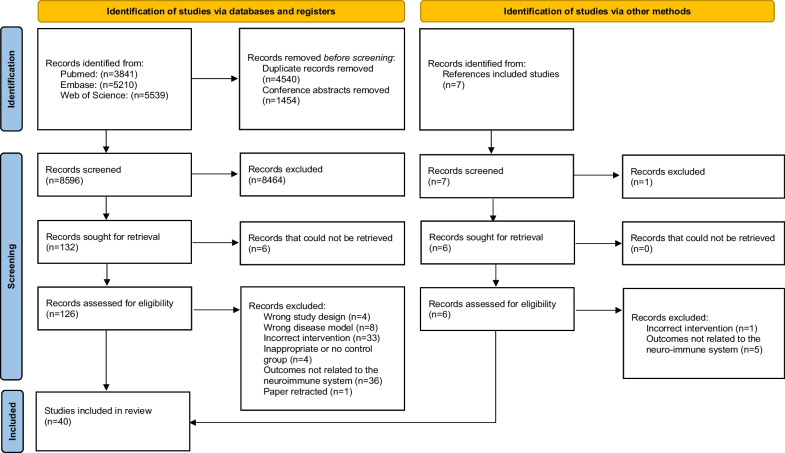
Flowchart of study selection

The literature search resulted in 14,590 records. After removal of duplicates and conference abstracts, 8596 records were screened. Following title and abstract screening, 132 articles remained. After full text screening of 126 retrieved papers, 40 studies were included in the review [20–59]. Reference screening yielded no additional included studies. The percentage agreement between the reviewers before deliberation was 75.8%. The flowchart of study selection is presented in Fig. 1.

### Study characteristics

The study characteristics of the 40 included studies are shown in Table 1. The studies included rats (26 studies) [21–29, 32–38, 40–42, 45–47, 51, 52, 56, 58], mice (13 studies) [20, 30, 31, 39, 44, 48–50, 53–55, 57, 59] or rabbits (1 study) [43]. Sprague Dawley rats (18 studies) [21, 24–29, 32, 35–38, 40, 42, 47, 51, 52, 56], C57BL/6J mice (8 studies) [20, 30, 31, 39, 48, 53, 57, 59] and Wistar rats (7 studies) [22, 23, 33, 34, 41, 45, 58] were the most frequently used animal strains. Thirty studies exclusively used male animals [20, 23, 25, 28–31, 33–39, 41–50, 52, 54–56, 58, 59]. Animal age ranged from 6 to 16 weeks, while age was specified as ‘adult’ in six studies [24, 34, 40, 45, 46, 51] and unclear in five studies [28, 29, 36, 44, 49].

**Table 1:** Study characteristics

Reference *Country*	Study design	Species *Strain*	Sex	Age (weeks)	Weight (g)	Disease model	Outcome measurement locations, side (if applicable)	Control group (*n*)	Intervention group (*n*)	Exercise start (dpi)	Length exercise program	Session duration	Intensity
Almeida C 2015 [44] *Brazil*	*RCT*	Mice *BALB/c*	M	NR	23.5 ± 0.24	CCI *Sciatic nerve, unilateral*	• Spinal cord dorsal horn L4–L6, ipsilateral • DRG L4 and L5, ipsilateral	SED (*n* = NR)	SE (*n* = 8)	7	5 weeks	Increasing from 10 min up to 50 min	NA
Arbat-Plana A 2015 [21] *Spain*	*ECT*	Rats *Sprague–Dawley*	F	8	250–300	Nerve transection^a^ *Sciatic nerve, unilateral*	Spinal cord segments L3–L6 (motoneuron pools), ipsilateral. For one outcome bilateral (ipsilateral data extracted)	SED (*n* = 16)	IG1: TR High intensity (*n* = 8)	3	5 days	Increasing: up to 60 min	Increasing: 10 cm/s up to 30 cm/sec
									IG2: TR Low intensity (*n* = 8)	3	2 weeks	2 × 30 min, 10 min rest	10 cm/s
Arbat-Plana A 2017A [26] *Spain*	*ECT*	Rats *Sprague–Dawley*	F	8	250–300	Nerve transection^a^ *Sciatic nerve, unilateral*	Spinal cord segments L3–L6 (motoneuron pools), ipsilateral. For one outcome bilateral (ipsilateral data extracted)	SED (*n* = 4)	IG1: TR High intensity (*n* = 4)	3	1.5 weeks	Increasing up to 60 min	10 cm/s, every 5 min + 2 cm/s to 30 cm/sec
									IG2: TR Low intensity (*n* = 4)	3		2 × 30 min, 10 min rest	10 cm/s
									IG3: VWR (*n* = 12)	0		voluntary	voluntary
Arbat-Plana A 2017B [27] *Spain*	*ECT*	Rats *Sprague–Dawley*	F	8	180–200	Nerve transection^a^ *Sciatic nerve, unilateral*	Spinal cord section L4–5, Bilateral (ipsilateral data extracted)	SED (*n* = 11)	TR (*n* = 11)	3	1.5 weeks	Increasing up to 60 min	10 cm/s, every 5 min + 2 cm/s until 30 cm/sec
Arbat-Plana A 2019 [32] *Spain*	*ECT*	Rats *Sprague–Dawley*	F	8	200–280	Nerve transection^a^ *Sciatic nerve, unilateral*	Spinal cord segments L3–L6, (motoneuron pools), ipsilateral. For one outcome bilateral (ipsilateral data extracted)	SED (*n* = 9)	TR (*n* = 9)	3	12 days	60 min	10 cm/s, every 5 min + 2 cm/s until 30 cm/sec
Ashour H 2017 [46] *Egypt*	*RCT*	Rats *Albino*	M	Adult, age unspecified	120–150	SNI *Sciatic nerve, unilateral*	• Serum • Fascia surrounding nerve bifurcation, ipsilateral • Gastrocnemius muscle, ipsilateral • Sciatic nerve, ipsilateral	SED (*n* = 8)	SE (*n* = 8)	NR	5 days	Increasing from 20 up to 90 min	NA
Bobinski F 2011 [54] *Brazil*	*ECT*	Mice *Swiss*	M	8–9	25–35	Nerve crush injury *Sciatic nerve, unilateral*	• Lumbar spinal cord (L1–L6), side not specified • Sciatic nerve, ipsilateral	SED (*n* = 8)	TR (*n* = 8)	3	2 weeks	30 min	10 m/min, 0% inclination
Bobinski F 2015 [55] *Brazil and USA*	*ECT*	Mice *Swiss*	M	8–9	25–35	Nerve crush injury *Sciatic nerve, unilateral*	Brainstem / Raphe Pallidus (RPa) / Raphe Magnus (RMg) / Raphe Obscurus (ROb)	SED (*n* = 8)	TR (*n* = 8)	3	2 weeks	30 min	10 m/min, 0% inclination
Bobinski F 2018 [49] *Brazil and USA*	*ECT*	Mice *Swiss* + *Balb/cJ wild type*	M	NR	20–30	Nerve crush injury *Sciatic nerve, unilateral*	• Lumbar spinal cord dorsal horn L1–L6 (specifically in laminae I–II and III–VI for some analyses), bilateral (ipsilateral data extracted) • Whole lumbar portion of the spinal cord L1–L6 for some analyses, side not specified • Sciatic nerve, ipsilateral	SED (*n* = 8)	TR (*n* = 8)	3	2 weeks	30 min	10 m/min, 0% inclination
Bonetti L 2016 [23] *Brazil*	*RCT*	Rats *Wistar*	M	12	280–330	Nerve crush injury *Sciatic nerve, unilateral*	Lumbar spinal cord dorsal horn L4–L6, laminae II–IV, ipsilateral	SED (*n* = 6)	TR (*n* = 6)	2	5 weeks	Increase: 20 up to 60 min	5 min 30% MET (5.5 m/min), 10–50 min 45–55% of MET (9 m/min), 5 min 30% MET
Byun Y 2005 [38] *South Korea*	*RCT*	Rats *Sprague–Dawley*	M	6	200 ± 10	Nerve crush injury *Sciatic nerve, unilateral*	Sciatic nerve, ipsilateral	SED (*n* = 8)	TR (*n* = 8)	3	12 days	30 min	8 m/min, 0% incline
Chen YW 2012 [29] *Taiwan*	*ECT*	Rats *Sprague–Dawley*	M	NR	250–300	CCI *Sciatic nerve, unilateral*	Sciatic nerve, ipsilateral	SED (*n* = 5)	IG1: SE (*n* = 5)	2	39 days	90 min	From 9 × 10 min to 1 × 90 min
									IG2: TR (*n* = 5)	2	6 weeks	Increasing from 15–30 min to 60 min	1st week 1.2 km/h, then 1.8 km/h
Cobianchi S 2013 [24] *Spain*	*RCT*	Rats *Sprague–Dawley*	F	Adult, age unspecified	240 ± 30	Nerve transection^a^ *Sciatic nerve, unilateral*	• Spinal cord ventral horn, ipsilateral • DRG L4–L5, ipsilateral	SED (*n* = 15)	TR (*n* = 8)	3	5 days	60 min	Increasing: 10 cm/s up to 32 cm/s
Coradini JG 2015 [33] *Brazil*	*RCT*	Rats *Wistar*	M	10.4	NR	CCI^b^ *Median nerve, unilateral*	Median nerve, ipsilateral	SED (*n* = 8)	SE (*n* = 8)	3	3 weeks	Increasing from 20 min up to 40 min	Overload of 10% body weight while swimming
Farzad B 2018 [34] *Iran*	*RCT*	Rats *Wistar*	M	Adult, age unspecified	180–220	CCI *Sciatic nerve, unilateral*	Spinal cord segments L4–L6, ipsilateral	SED (*n* = 7)	SE (*n* = 7)	3	4 weeks	Increasing duration from bouts of 10 min up	NA
Huang PC 2017 [28] *Taiwan*	*RCT*	Rats *Sprague–Dawley*	M	NR	220–270	CCI *Sciatic nerve, unilateral*	Sciatic nerve, side not specified	SED (*n* = 22)	TR (*n* = 22)	8	3 weeks	30 min	14–16 m/min, 8% incline
Hung CH 2016 [36] *Taiwan*	*RCT*	Rats *Sprague–Dawley*	M	NR	220–270	CCI *Sciatic nerve, unilateral*	• Spinal cords L4–L5, side not specified • Spinal cords dorsal horn L4–L5, ipsilateral	SED (*n* = 10)	TR (*n* = 10)	3	4 weeks	30 min	14 -16 m/min, 8% incline
Kami K 2016A [20] *Japan*	*ECT*	Mice *C57BL/6 J*	M	10	NR	PSL *Sciatic nerve, unilateral*	Brain, lVTA,, bilateral (contralateral data extracted)	SED (*n* = 5)	VWR (*n* = 5)	NR	15 days	voluntary	voluntary
Kami K 2016B [30] *Japan*	*ECT*	Mice *C57BL/6 J*	M	12	NR	PSL *Sciatic nerve, unilateral*	Lumbar spinal cord dorsal horn L4–5, bilateral (ipsilateral data extracted)	SED (*n* = 6)	TR (*n* = 6)	2	5 days	60 min	7 m/min
Kami K 2016C [31] *Japan*	*ECT*	Mice *C57BL/6 J*	M	13	NR	PSL *Sciatic nerve, unilateral*	Lumbar spinal cord dorsal horn L4–5, bilateral (ipsilateral data extracted)	SED (*n* = 6)	TR (*n* = 6)	2	5 days	60 min	7 m/min
Kim JE 2020 [37] *Korea*	*ECT*	Rats *Sprague–Dawley*	M	6	160	Nerve crush injury *Sciatic nerve, unilateral*	Sciatic nerve, ipsilateral	CG1: SED 7 days (*n* = 10)	IG1: TR 7 days (*n* = 10)	3	1 week	20 min	8 m/min
								CG2: SED 14 days (*n* = 10)	IG2: TR 14 days (*n* = 10)	3	2 weeks	20 min	8 m/min
Kim S 2013 [35] *Korea*	*RCT*	Rats *Sprague–Dawley*	M	4	NR	Nerve transection *Sciatic nerve, unilateral*	• Serum • Liver • Gastrocnemius, soleus and tibialis anterior muscles, side unspecified	SED (*n* = 6)	TR (*n* = 6)	7	4 weeks	25–30 min	15 m/min, 3–5% inclination
Kim YJ 2015 [25] *Korea*	*RCT*	Rats *Sprague–Dawley*	M	10	250–300	CCI *Sciatic nerve, unilateral*	• Rostral ventral medulla • Lumbar spinal cords (2 cm long), side unspecified	SED (*n* = 11)	TR (*n* = 11)	NR	4 weeks	40 min Warm-up 5 min; Main exercise 30 min; Cool-down: 5 min	8 m/min for 5 min, 11 m/min for 5 min, 22 m/min for 20 min
Korb A 2010 [41] *Brazil*	*RCT*	Rats *Wistar*	M	16	200–250	Nerve transection *Sciatic nerve, unilateral*	• Magnus raphe nucleus • Dorsal raphe nucleus • Lumbosacral ventral horn of spinal cord, bilateral (ipsilateral data extracted) • Lumbosacral spinal cord dorsal horn, bilateral (ipsilateral data extracted)	SED (*n* = 5)	TR (*n* = 5)	7	4 weeks	20–60 min	30% of MET (5 min 5,5 m/min), 45–55% of MET (10-50 min at ~ 9 m/min), 30% of MET (5 min at 5,5 m/min)
Krakowiak J 2015 [53] *USA*	*ECT*	Mice *C57BL/6 J wild type*	F	> 8	NR	Nerve transection *Sciatic nerve, unilateral*	Spinal cord segments L3–L6, (motoneurons), ipsilateral	SED (*n* = 8)	TR (*n* = 9)	3	2 weeks	28 min	20 m/min, 4 × 2 min/5 min rest
Liao CF 2017 [42] *Taiwan*	*RCT*	Rats *Sprague–Dawley*	M	9	201–225	Nerve transection^a^ *Sciatic nerve, unilateral*	Spinal cord L4, ipsilateral	SED (*n* = 10)	IG1: SE10 (*n* = 10)	7	3 weeks	10 min	NA
									IG2: SE20 (*n* = 10)	7	3 weeks	20 min	NA
									IG2: SE30 (*n* = 10)	7	3 weeks	30 min	NA
Liu C 2014 [57] *China and USA*	*RCT*	Mice *C57BL/6 J*	M + F	> 2 months	18–32	Nerve transection *Sciatic nerve, unilateral*	Spinal cord segments L3–L5, (motoneurons), ipsilateral	CG1: SED Male (*n* = 4)	IG1: TR Male Continuous (*n* = 4)	3	2 weeks	60 min	10 m /min
								CG2: SED Female (*n* = 4)	IG2: TR Female Continuous (*n* = 4)	3	2 weeks	60 min	10 m /min
								CG1: SED Male (*n* = 4)	IG3: TR Male Interval (*n* = 4)	3	2 weeks	28 min: 4 × 2 min + 5 min rest	20 m / min
								CG2: SED Female (*n* = 4)	IG4: TR Female Interval (*n* = 4)	3	2 weeks	28 min: 4 × 2 min + 5 min rest	20 m / min
Lopes BC 2020 [58] *Brazil*	*ECT*	Rats *Wistar*	M	8	280 ± 20	CCI *Sciatic nerve, unilateral*	• Cerebral cortex • Brainstem • Spinal cord, level and side unspecified	SED (*n* = 6)	TR (*n* = 6)	15	8 days	20 min	70% VO2max, 0° incline
Lopez-Alvarez VM 2015 [40] *Spain*	*RCT*	Rats *Sprague–Dawley*	F	Adult, age unspecified	240 ± 30	Nerve transection^a^ *Sciatic nerve, unilateral*	• Epidermal and subepidermal nerve fibers from footpad mid-plan skin sections, hind paws, ipsilateral. For 1 outcome bilateral (ipsilateral data extracted) • Spinal cord segments L3–6, for some outcome bilateral (ipsilateral data extracted) • Spinal cord dorsal horn L3 and L5, ipsilateral • Dorsal Root Ganglion L3–L6, bilateral (ipsilateral data extracted) • For one outcome Dorsal Root Ganglion L3, ipsilateral	SED (*n* = 8)	IG1: TR1 (*n* = 8)	3	5 days (day 3–7)	60 min	10 cm/s, + 2 cm/s every 5 min until 32 cm/s
									IG2: TR2 (*n* = 7)	3	5 days (day 10–14)	60 min	
Lopez-Alvarez VM 2018 [51] *Spain*	*RCT*	Rats *Sprague–Dawley*	F	Adult, age unspecified	240 ± 30	Nerve transection^a^ *Sciatic nerve, unilateral*	• Brain, projection areas of injured side • Lumbar spinal cord dorsal horn segments L4–L5, bilateral (ipsilateral data extracted)	SED (*n* = 8)	TR (*n* = 10)	3	12 days	60 min	10 cm/s, + 2 cm/s every 5 min until 32 cm/s
Martins DF 2018 [50] *Brazil*	*ECT*	Mice *Swiss*	M	8	25–30	Nerve crush injury *Sciatic nerve, unilateral*	• Sciatic nerve, ipsilateral • Gastrocnemius and soleus muscles, side not specified	SED (*n* = 8)	TR (*n* = 8)	7	8 weeks	30 min	14 m/min, − 16° slope (downhill running)
Park JS 2014 [59] *USA*	*RCT*	Mice *C57BL/6 J*	M	6–8	20–30	Nerve transection^a^ *Median and ulnar nerves, unilateral*	• Median nerve, ipsilateral • Forearm extrinsic finger flexor muscles, side unspecified • Serum	SED (*n* = 8)	TR (*n* = 8)	4	6 weeks	60 min	10 m /min, 0° incline, 5 min warm up and cooldown at 6 m/min
Safakhah HA 2017 [45] *Iran*	*RCT*	Rats *Wistar*	M	Adult, age unspecified	220 ± 20	CCI *Sciatic nerve, unilateral*	• Cerebrospinal fluid from cisterna magna • Serum	SED (*n* = 6–9)	TR (*n* = 6–9)	4	3 weeks	30 min	16 m/min
Sartini S 2013 [52] *Italy*	*ECT*	Rats *Sprague–Dawley*	M	6	150–200	Nerve crush injury *Soleus nerve, unilateral*	Soleus muscle, ipsilateral	SED (*n* = 5)	TR (*n* = 5)	4	Outcome 10 dpi: 7 days 40 dpi: 37 days	30 min, twice per day	5 min on, 5 min off, on periods were 4 min increase from 0–27 m/min and 1 min at 27 m/min
Sheahan TD 2015 [39] *USA*	*ECT*	Mice *C57BL/6 J*	M	7–10	21–24.6	SNI *Sciatic nerve, unilateral*	Center hindpaw Intraepidermal nerve fiber density, ipsilateral	CG1: SED 2 h/night (*n* = 7)	IG1: VWR 2 h/night (*n* = 8)	8–10	6 days	2 h/night	Voluntary
								CG2: SED 12 h/night (*n* = 5)	IG2: VWR 12 h/night (*n* = 7)	8–10	6 days	12 h/night	Voluntary
Sumizono M 2018 [56] *Japan*	*RCT*	Rats *Sprague–Dawley*	M	8	274.3 ± 21.2	CCI *Sciatic nerve, unilateral*	• Periaqueductal gray • Lumbar spinal cord dorsal horn segments, ipsilateral	SED (*n* = 25)	IG1: TR1 High frequency (*n* = 27)	1 or 2	5 weeks	30 min	TR1: 5 day per week 20 m/min
									IG2: TR2 Low frequency (*n* = 10)	1 or 2	5 weeks	30 min	TR2: 3 day per week 20 m/min
Taguchi S 2015 [48] *Japan*	*ECT*	Mice *C57BL/6 J*	M	12	NR	PSL *Sciatic nerve, unilateral*	Sciatic nerve, ipsilateral	SED (*n* = 8)	TR (*n* = 8)	2	5 days	60 min	7 m/min
Tsai KL 2017 [47] *Taiwan*	*ECT*	Rats *Sprague–Dawley*	M	8–9	285–335	CCI *Sciatic nerve, unilateral*	Sciatic nerve, ipsilateral	SED (*n* = 6)	IG1: TR0% (*n* = 6)	6	3 weeks	30 min	14–16 m/min, 0% incline
									IG2: TR8% (*n* = 6)	6	3 weeks	30 min	14–16 m/min, 8% incline
Wang Y 2016 [43] *China*	*RCT*	Rabbits *New Zealand white*	M	11–12	1780 ± 120	Nerve crush injury *Sciatic nerve, unilateral*	Tibia, cortical bone from the tibial shaft, ipsilateral	SED (*n* = 6)	TR (*n* = 6)	3	4 weeks	20 min	Day 1–3: 10 m/min, day 4–6: 15 m/min, 7 days + : 20 m/min
Yamaoka S [22] *Japan*	*RCT*	Rats *Wistar*	F	6–8	NR	Spinal nerve ligation *L5, unilateral*	Spinal cord dorsal horn, resected Th11, bilateral (ipsilateral data extracted)	SED (*n* = 6)	TR (*n* = 6)	1	NR	10 min	Increasing: 10 m/min up to 20 m/min

*CCI* chronic constriction injury, *CG* control group, *dpi* days post-injury, *ECT* experimentally controlled trial, *F* female, *IG* intervention group, *M* male, *MET* maximum exercise test, *NA* not applicable, *NR* not reported, *PSL* partial sciatic nerve ligation, *RCT* randomized controlled trial, *SE* swimming exercise, *SED* sedentary, *SNI* spared nerve injury, *TR* treadmill running, *USA* United States of America, *VWR* voluntary wheel running

^a^Cut and repair

^b^Vehicle injection

Almost all studies involved the sciatic nerve (36 studies) [20, 21, 23–32, 34–51, 53–58]; other studies used the soleus nerve (the soleus nerve branch off the tibial nerve) (1 study)[52], median and ulnar nerve (1 study) [59], median nerve (1 study) [33] and the L5 spinal nerve (1 study) [22].

A variety of disease models was used. Nerve transection was performed in 13 studies [21, 24, 26, 27, 32, 35, 40–42, 51, 53, 57, 59], of which nine also used a form of repair [21, 24, 26, 27, 32, 40, 42, 51, 59]. The chronic constriction injury (CCI) model was used in 11 studies [25, 28, 29, 33, 34, 36, 44, 45, 47, 56, 58], a nerve crush injury in nine studies [23, 37, 38, 43, 49, 50, 52, 54, 55], partial sciatic nerve ligation (PSL) was performed in four studies [20, 30, 31, 48], spared nerve injury (SNI) was used in two studies [39, 46] and one study used spinal nerve ligation (SNL) [22].

The exercise programs reported in the studies were treadmill running (34 studies) [20–32, 36–41, 43, 45, 47–59], swimming (6 studies) [29, 33, 34, 42, 44, 46] and voluntary wheel running (3 studies) [20, 26, 39]. The timing of the start of the exercise programs varied from immediately (0 days) to 15 days after injury. Three studies [20, 25, 46] did not report the starting day of the exercise program. The length of the exercise program varied from 5 days to 8 weeks. One study [22] did not report the length of the exercise program.

### Risk of bias

**Fig. 2:**
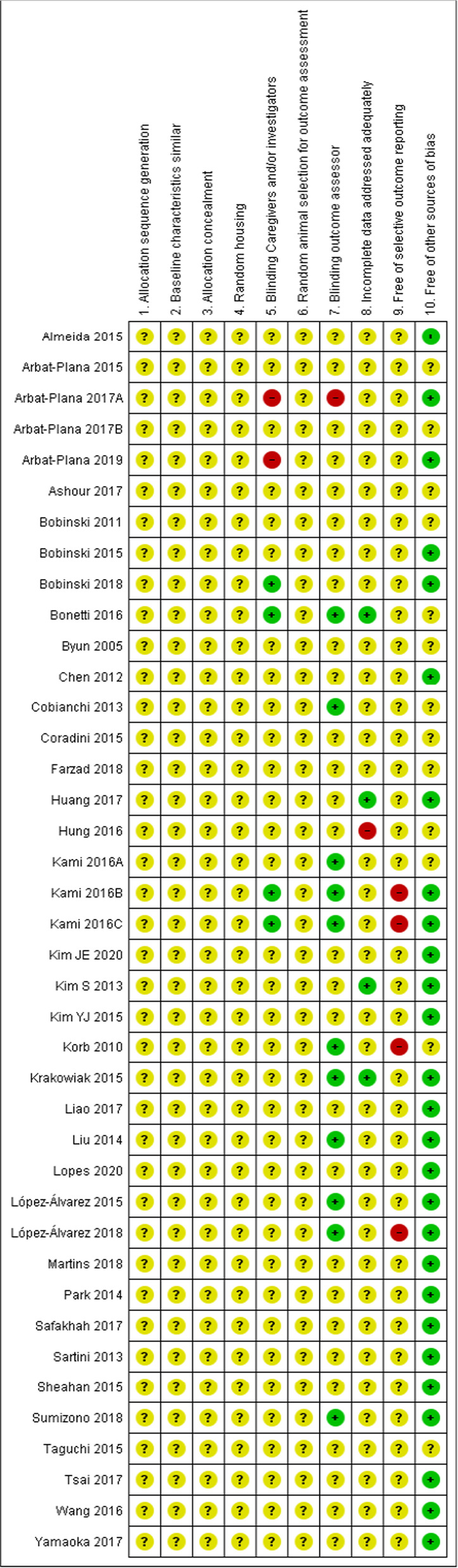
Risk of bias assessment. A ‘ + ’ represents low risk of bias, a ‘−’ high risk of bias and ‘?’ unclear risk of bias

The results of the risk of bias assessment are shown in Fig. 2. Overall, 86.5% of the criteria were marked ‘unclear’, because essential information regarding the methodology was missing. For five criteria, all studies scored ‘unclear’, namely allocation sequence generation and application, similarity of the groups at baseline, adequate allocation concealment, random housing of animals and random selection of animals for outcome assessment. Four studies successfully blinded the caregivers and investigators [23, 30, 31, 49], 11 studies adequately blinded the outcome assessor [20, 23, 24, 30, 31, 40, 41, 51, 53, 56, 57], four studies adequately addressed incomplete data [23, 28, 35, 53] and 27 studies were deemed ‘free of other problems that could result in high risk of bias’ [22, 25, 26, 28–32, 35, 37, 39, 40, 42–45, 47, 49–53, 55–59]. Four studies were considered not to be free of selective outcome reporting [30, 31, 41, 51]. Given the high number of ‘unclear’ scores, none of the studies were deemed to have a low risk of bias. The percentage agreement for the risk of bias assessment between the reviewers was 87.8%.

### Results of syntheses

**Fig. 3:**
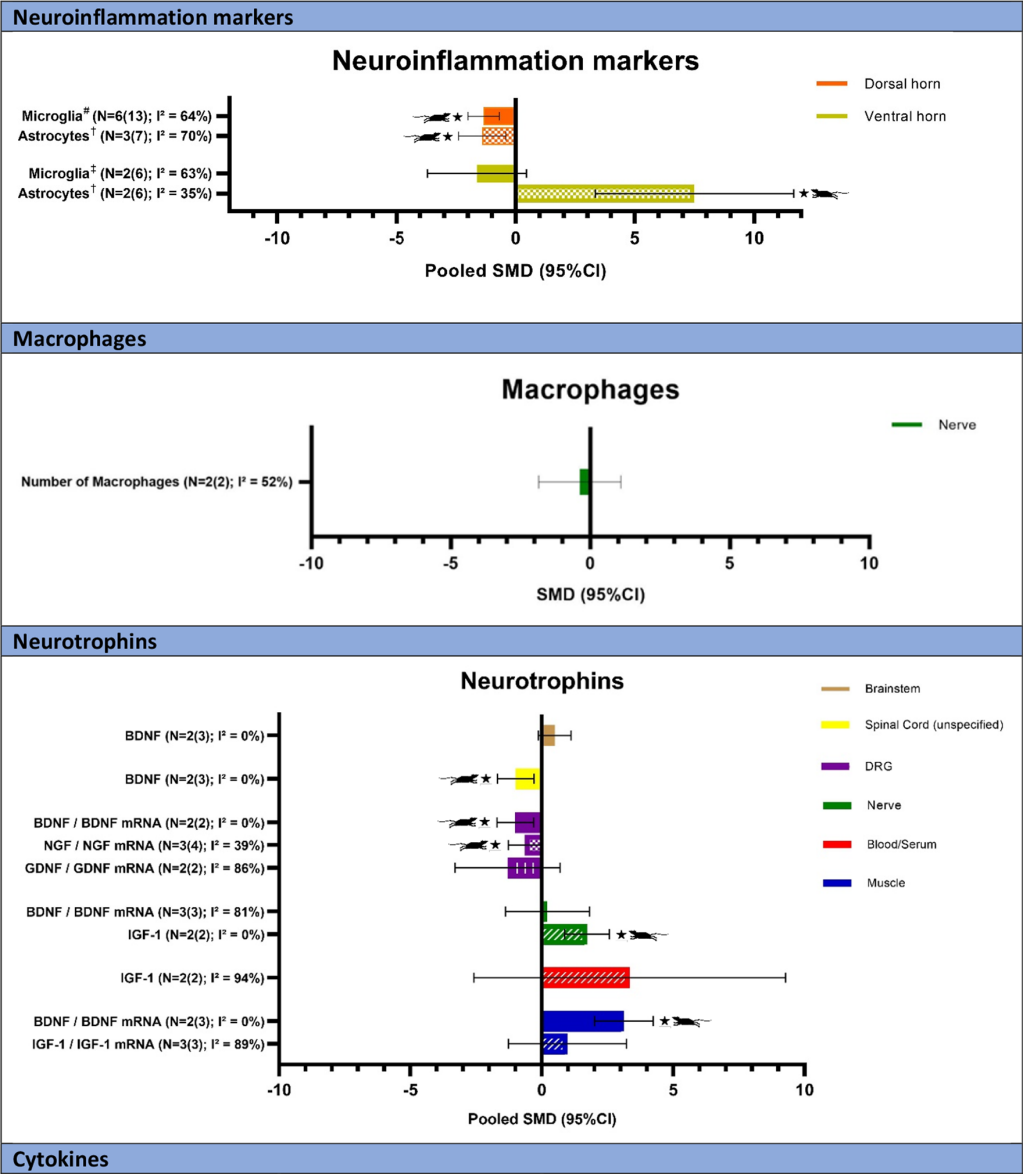

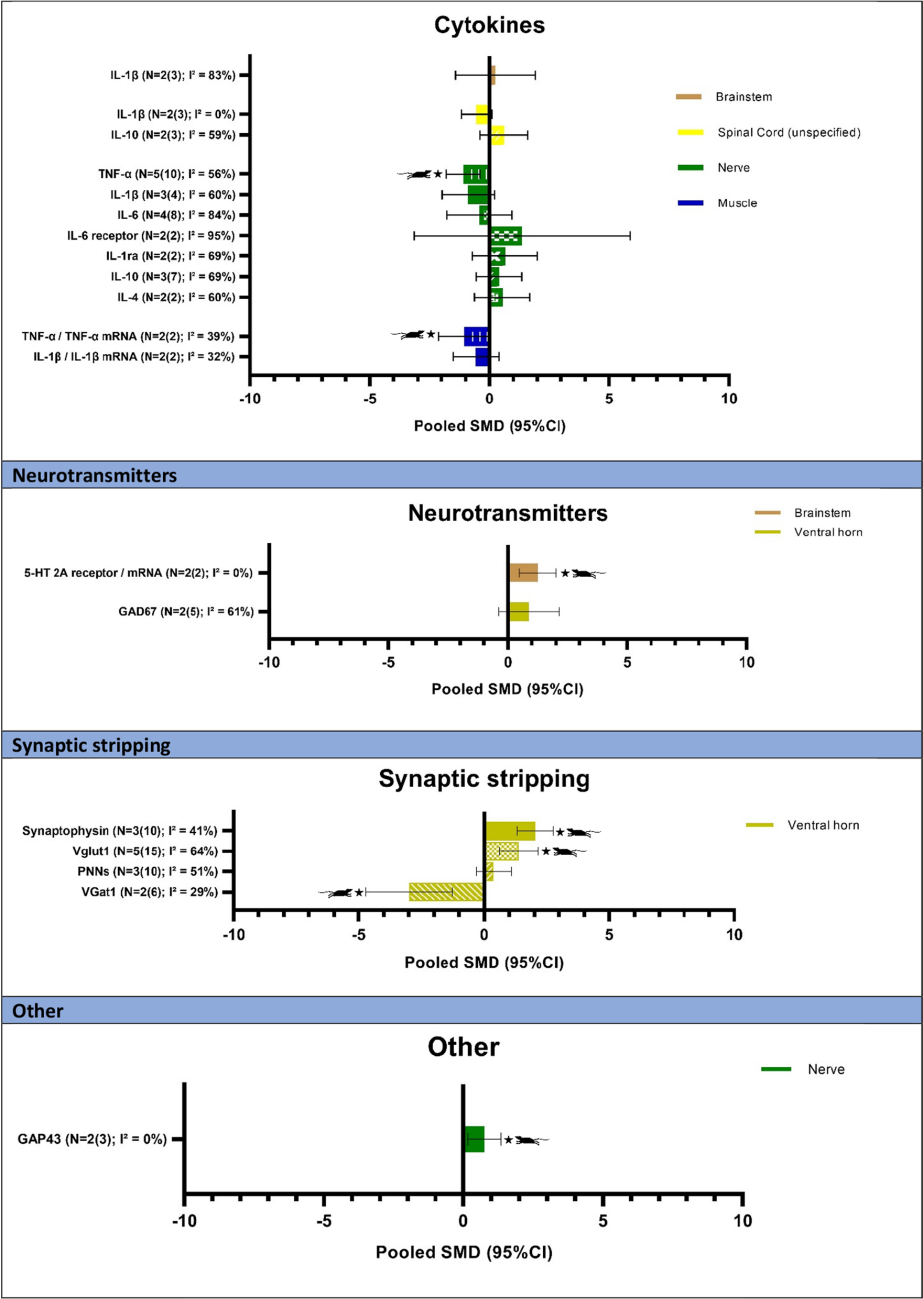
Results meta-analyses. *N* = number of studies (number of comparisons). * *p* < 0.05; ^#^ Iba1 or CD^−^11b^+^; †GFAP; ^e^ Iba1;  Results in favor of exercised animals compared to controls. *BDNF* brain-derived neurotrophic factor, *GAD67* glutamic acid decarboxylase 67, *GAP43* Growth Associated Protein 43, *GDNF* glial cell line-derived neurotrophic factor, *IGF-1* insulin-like growth factor-1, *IL* Interleukin, *NGF* nerve growth factor, *PNN* perineuronal net, *SMD* standardized mean difference, *TNF-α* tumor necrosis factor-α, *VGat1* vesicular GABA transporter 1, *VGlut1* vesicular glutamate transporter 1

An overview of all neuroimmune responses and the number of studies and comparisons that are available is shown in Table 2. In total, 139 comparisons of neuroimmune responses have been studied, which have been organized per class of neuroimmune substance and analyzed according to anatomical location. In total, 43 meta-analyses could be performed. Results for the meta-analyses per class are reported below and can be found in Table 2 and Fig. 3. An overview of all forest plots for the meta-analyses can be found in Additional file 2. Forest plots for comparisons for which a meta-analysis could not be conducted can be found in Additional file 3.

**Table 2:** Overview all neuroimmune responses

	Number of original studies (*N*)	Total number of comparisons (*N*)	Pooled SMD (if applicable)	Favors
*Neuroinflammation markers*				
Brainstem				
1.1.1 Microglia (Iba1)	1	1	N.A. (see Additional file 3 C1)	
Dorsal horn				
1.2.1 Microglia (Iba1 or CD^−^11b^+^)	6	13	**− 1.34 [− 1.99, − 0.68]**	EXP
1.2.2 BDNF + Iba1	1	3	N.A. (see Additional file 3 C1)	
1.2.3 Astrocytes (GFAP)	3	7	**− 1.40 [− 2.39, − 0.41]**	EXP
1.2.4 HDAC + nuclei	1	1	N.A. (see Additional file 3 C1)	
Ventral horn (Motoneurons)				
1.3.1 Microglia^a^ (Iba1)	2	6	− 1.62 [− 3.70, 0.45]	NS
1.3.2 Astrocytes^a^ (GFAP)	2	6	**7.50 [3.33, 11.68]**	EXP
Dorsal root ganglion				
1.4.1 Astrocytes	1	1	N.A. (see Additional file 3 C1)	
*Macrophages*				
Spinal cord (unspecified)				
2.1.1 Macrophage density	1	3	N.A. (see Additional file 3 C2)	
Nerve				
2.2.1 Number of macrophages (F4/80)	2	2	− 0.38 [− 1.85, 1.09]	NS
2.2.2 Number of CD68 + macrophages	1	1	N.A. (see Additional file 3 C2)	
2.2.3 Number of CD206 + macrophages	1	1	N.A. (see Additional file 3 C2)	
2.2.4. Relative proportion M1 Macrophages^b^ (% of total macrophages)	1	1	N.A. (see Additional file 3 C2)	
2.2.5. Relative proportion M2 Macrophages^b^ (% of total macrophages)	1	1	N.A. (see Additional file 3 C2)	
2.2.6. Relative proportion Intermediate Macrophages^b^ (% of total macrophages)	1	0	N.A	
*Neurotrophins*				
Brain				
A) Cerebral cortex 3.1.1 BDNF	1	2	N.A. (see Additional file 3 C3)	
B) Brainstem 3.2.1 BDNF	2	3	0.49 [− 0.13, 1.11]	NS
Spinal cord (unspecified)				
3.3.1 BDNF	2	3	**− 0.99 [− 1.69, − 0.29]**	EXP
3.3.4 β-NGF	1	1	N.A. (see Additional file 3 C3)	
Dorsal horn				
3.4.1 BDNF	1	5	N.A. (see Additional file 3 C3)	
3.4.3 NT3	1	1	N.A. (see Additional file 3 C3)	
Ventral horn				
3.5.1 BDNF mRNA	1	1	N.A. (see Additional file 3 C3)	
3.5.2 NT3 mRNA	1	1	N.A. (see Additional file 3 C3)	
3.5.3 NGF mRNA	1	1	N.A. (see Additional file 3 C3)	
3.5.4 GDNF mRNA	1	1	N.A. (see Additional file 3 C3)	
3.5.5 TrkB receptor	1	1	N.A. (see Additional file 3 C3)	
3.5.6 TrkC receptor	1	1	N.A. (see Additional file 3 C3)	
3.5.7 p-AKT/AKT	1	1	N.A. (see Additional file 3 C3)	
Dorsal root ganglion				
3.6.1 BDNF / BDNF mRNA	2	2	**− 1.01 [− 1.70, − 0.31]**	EXP
3.6.2 NGF / NGF mRNA	3	4	**− 0.64 [− 1.29, 0.00]**	EXP
3.6.3 GDNF / GDNF mRNA	2	2	− 1.30 [− 3.30, 0.69]	NS
3.6.4 NT3 mRNA	1	1	N.A. (see Additional file 3 C3)	
Nerve				
3.7.1 BDNF / BDNF mRNA	3	3	0.21 [− 1.38, 1.81]	NS
3.7.2 GDNF	1	1	N.A. (see Additional file 3 C3)	
3.7.3 IGF-1	2	2	**1.72 [0.87, 2.57]**	EXP
Blood/serum				
3.8.1 BDNF^c^	1	0	N.A	
3.8.2 GDNF	1	1	N.A. (see Additional file 3 C3)	
3.8.3 IGF-1	2	2	3.35 [− 2.59, 9.28]	NS
Muscle				
3.9.1 BDNF	2	3	**3.12 [2.00, 4.24]**	EXP
3.9.2 GDNF	1	1	N.A. (see Additional file 3 C3)	
3.9.3 IGF-1 / IGF-1 mRNA^d^	3	3	0.97 [− 1.27, 3.22]	NS
3.9.4 TrkB kinase	1	1	N.A. (see Additional file 3 C3)	
3.9.5 p-TrkB receptors	1	1	N.A. (see Additional file 3 C3)	
Liver				
3.10.1 IGF-1 mRNA	1	1	N.A. (see Additional file 3 C3)	
*Cytokines*				
Cerebral cortex				
4.1.1 IL-1β	1	2	N.A. (see Additional file 3 C4)	
4.1.2 IL-4	1	2	N.A. (see Additional file 3 C4)	
Brainstem				
4.2.1 TNF-α	1	1	N.A. (see Additional file 3 C4)	
4.2.2 IL-1β	2	3	0.24 [− 1.43, 1.91]	NS
4.2.3 IL-4	1	2	N.A. (see Additional file 3 C4)	
Spinal cord (unspecified)				
4.3.1 TNF-α	1	1	N.A. (see Additional file 3 C4)	
4.3.2 IL-1β	2	3	− 0.54 [− 1.18, 0.10]	NS
4.3.4 IL-4	1	2	N.A. (see Additional file 3 C4)	
4.3.6 IL-6	1	2	N.A. (see Additional file 3 C4)	
4.3.7 IL-6 receptor	1	1	N.A. (see Additional file 3 C4)	
4.3.8 IL-10	2	3	0.60 [− 0.39, 1.60]	NS
4.3.9 Irisin	1	1	N.A. (see Additional file 3 C4)	
Dorsal horn				
4.4.1 IL-1 receptor antagonist	1	1	N.A. (see Additional file 3 C4)	
4.4.2 IL-4	1	1	N.A. (see Additional file 3 C4)	
4.4.3 IL-5	1	1	N.A. (see Additional file 3 C4)	
4.4.4 IL-6	1	1	N.A. (see Additional file 3 C4)	
Nerve				
4.5.1 TNF-α	5	10	**-1.10 [− 1.81, − 0.39]**	EXP
4.5.2 IL-1β	3	4	− 0.89 [− 1.98, 0.21]	NS
4.5.3 IL-1 receptor antagonist	2	2	0.64 [− 0.70, 1.99]	NS
4.5.4 IL-4	2	2	0.54 [− 0.62, 1.69]	NS
4.5.5 IL-5	1	1	N.A. (see Additional file 3 C4)	
4.5.6 IL-6	4	8	− 0.42 [− 1.78, 0.93]	NS
4.5.7 IL-6 receptor	2	2	1.36 [− 3.16, 5.88]	NS
4.5.8 STAT3	1	1	N.A. (see Additional file 3 C4)	
4.5.9 IL-10	3	7	0.40 [− 0.54, 1.35]	NS
Blood/serum				
4.6.1 IL-6	1	1	N.A. (see Additional file 3 C4)	
Muscle				
4.7.1 TNF-α	2	2	**− 1.06 [− 2.12, − 0.01]**	EXP
4.7.2 IL-1β	2	2	− 0.57 [− 1.52, 0.39]	NS
4.7.3 IL-1ra	1	1	N.A. (see Additional file 3 C4)	
4.7.4 IL-4	1	1	N.A. (see Additional file 3 C4)	
4.7.5 IL-6	1	1	N.A. (see Additional file 3 C4)	
Cerebrospinal fluid				
4.8.1 TNF-α	1	1	N.A. (see Additional file 3 C4)	
*Neurotransmitters*				
(nor)adrenergic				
Brainstem				
5.1.1 α1a receptor	1	1	N.A. (see Additional file 3 C5)	
5.1.2 β2 receptor	1	1	N.A. (see Additional file 3 C5)	
Dorsal horn				
5.2.1 α1a receptor	1	1	N.A. (see Additional file 3 C5)	
5.2.2 β2 receptor	1	1	N.A. (see Additional file 3 C5)	
Serotonin				
Brainstem				
6.1.1 5-HT (unspecified)^e^	1	1	N.A. (see Additional file 3 C6)	
6.1.2 5-HT 1A receptor	1	1	N.A. (see Additional file 3 C6)	
6.1.3 5-HT 1B receptor	1	1	N.A. (see Additional file 3 C6)	
6.1.4 5-HT 2A receptor/receptor mRNA	2	2	**1.25 [0.49, 2.01]**	EXP
6.1.5 5-HT 2C receptor	1	1	N.A. (see Additional file 3 C6)	
6.1.6 5-HT 3A receptor	1	1	N.A. (see Additional file 3 C6)	
6.1.7 5-HT 7 receptor	1	1	N.A. (see Additional file 3 C6)	
6.1.8 SERT (serotonin transporter)	1	1	N.A. (see Additional file 3 C6)	
6.1.9 Tryptophan hydroxylase 2 (Tph2)	1	1	N.A. (see Additional file 3 C6)	
Dorsal horn				
6.2.1 5-HT receptor (unspecified)	1	1	N.A. (see Additional file 3 C6)	
6.2.2 5-HT 2A receptor	1	1	N.A. (see Additional file 3 C6)	
Ventral horn				
6.3.1 5-HT receptor (unspecified)	1	1	N.A. (see Additional file 3 C6)	
GABA				
SPINAL CORD (unspecified)				
7.1.1 GAD65	1	1	N.A. (see Additional file 3 C7)	
Dorsal horn				
7.2.1 GABA	1	1	N.A. (see Additional file 3 C7)	
7.2.2 GABA + /NeuN + neurons	1	1	N.A. (see Additional file 3 C7)	
7.2.4 GAD65/67 (no distinction)	1	1	N.A. (see Additional file 3 C7)	
7.2.6 Rnf34	1	3	N.A. (see Additional file 3 C7)	
Ventral horn				
7.1.5 GAD67	2	5	0.88 [-0.38, 2.13]	NS
Dopamine				
Brainstem				
8.1.1 TH immunoreactivity	1	1	N.A. (see Additional file 3 C8)	
Purine				
Spinal cord (unspecified)				
9.1.1 P2X3 (ATP receptor)	1	1	N.A. (see Additional file 3 C9)	
Opioid system				
Brainstem				
10.1.1 μ-opioid receptor	1	1	N.A. (SEE Additional file 3 C10)	
10.1.2 β-endorphin	1	5	N.A. (see Additional file 3 C10)	
Spinal cord (unspecified)				
10.2.1 μ-opioid receptor	1	1	N.A. (see Additional file 3 C10)	
Dorsal horn				
10.3.1 μ-opioid receptor	1	5	N.A. (see Additional file 3 C10)	
*Neuropeptides*				
Dorsal horn				
11.1.1 CGRP	1	3	N.A. (see Additional file 3 C11)	
11.1.2 PACAP mRNA	1	3	N.A. (see Additional file 3 C11)	
Bone				
11.4.1 Substance P	1	1	N.A. (see Additional file 3 C11)	
(Sub)epidermis				
11.5.1 Intraepidermal nerve fiber density	1	2	N.A. (see Additional file 3 C11)	
11.5.2 CGRP	1	3	N.A. (see Additional file 3 C11)	
11.5.4 PGP	1	3	N.A. (see Additional file 3 C11)	
*Synaptic stripping*				
Dorsal horn				
12.1.1 Synaptophysin	1	1	N.A. (see Additional file 3 C12)	
Ventral horn (motoneurons)				
12.2.1 Synaptophysin^e,f^	3	10	**2.05 [1.32, 2.77]**	EXP
12.2.2 Vglut1^e,f^	5	15	**1.38 [0.62, 2.15]**	EXP
12.2.3 Gephyrin	1	4	N.A. (see Additional file 3 C12)	
12.2.4 Perineuronal Nets^e^	3	10	0.39 [− 0.31, 1.09]	NS
12.2.5 VGat1^e^	2	6	**− 2.99 [− 4.72, − 1.26]**	EXP
C-Boutons	1	5	N.A. (see Additional file 3 C12)	
*Other*				
Potassium-chloride cotransporters (KCC)				
Dorsal horn				
13.1.1 KCC2^e^	1	2	N.A. (see Additional file 3 C13)	
13.1.2 pKCC2	1	2	N.A. (see Additional file 3 C13)	
Dorsal root ganglion				
13.2.1 NKCC1	1	2	N.A. (see Additional file 3 C13)	
13.2.2 pNKCC1	1	2	N.A. (see Additional file 3 C13)	
MAPK signaling pathway				
Nerve				
14.1.1 p-ERK1/2	1	2	N.A. (see Additional file 3 C14)	
14.1.2 p-38MAPK	1	2	N.A. (see Additional file 3 C14)	
14.1.3 c-Jun N-terminated kinase (JNK)	1	2	N.A. (see Additional file 3 C14)	
14.1.4 p–c-Jun	1	2	N.A. (see Additional file 3 C14)	
14.1.5 ATF3	1	2	N.A. (see Additional file 3 C14)	
GAP43				
Dorsal root ganglion				
15.1.1 CGRP + GAP43	1	1	N.A. (see Additional file 3 C15)	
Nerve				
15.2.1 GAP43	2	3	**0.76 [0.17, 1.35]**	EXP
(Sub)epidermis				
15.3.1 CGRP + GAP43^g^	1	3	N.A. (see Additional file 3 C15)	
CREB				
Brainstem				
16.1.1 p-CREB	1	1	N.A. (see Additional file 3 C16)	
15.1.2 p-CREB/TH +	1	1	N.A. (see Additional file 3 C16)	
Dorsal horn				
16.2.1 p-CREB	1	1	N.A. (see Additional file 3 C16)	
16.2.2 total CREB	1	1	N.A. (see Additional file 3 C16)	
Nerve				
16.3.1 p-CREB	1	2	N.A. (see Additional file 3 C16)	
Oxidative stress				
Serum				
17.2.1 FRAP	1	1	N.A. (see Additional file 3 C17)	
17.2.2 MDA	1	1	N.A. (see Additional file 3 C17)	
PLCy-1				
Dorsal horn				
18.1.1 phosphorylated PLCy-1	1	1	N.A. (see Additional file 3 C18)	
18.1.2 total PLCy-1	1	1	N.A. (see Additional file 3 C18)	
Heat shock protein 72				
Nerve				
Heat shock protein 72^h^	1	0	N.A	

*EXP* favors experimental, *NS* not significant

Bold values represent significant results. ^a^Unable to include two studies due to inability to determine mean and/or SD for all groups [21, 27]. ^b^Unable to include one study due to inability to determine mean and/or SD for all groups [48]. ^c^Unable to include one study due to inability to determine mean and/or SD for all groups [59]. ^d^Sensitivity analyses performed, due to the same but opposite effects reported in two muscles. ^e^Unable to include one comparison due to inability to determine SD for all groups [41]. ^e^Unable to include one study in meta-analysis due to inability to determine mean and/or SD for all groups [27]. ^f^Added a negative sign before results for Arbat-Plana [21, 26], because unit of measurement was % loss. ^g^Unable to include two comparisons from one study due to inability to determine mean and/or SD for all groups [40]. ^h^Unable to include two comparisons from one study due to inability to determine mean and/or SD for all groups [29]

#### Neuroinflammation markers

##### Dorsal horn

In the dorsal horn, microglia (Iba1 or CD^−^11b^+^) markers (6 studies, 13 comparisons (*N* = 6; cf = 13), pooled SMD: − 1.34 (95%CI: − 1.99, − 0.68)) [30, 36, 40, 44, 49, 56] and astrocyte (GFAP) marker levels ((*N* = 3; cf = 7), pooled SMD: − 1.40 (95%CI: − 2.39, − 0.41)) [30, 49, 56] were significantly lower compared to controls.

##### Ventral horn

Microglia (Iba1) and astrocyte (GFAP) reactivity surrounding axotomized motoneurons was determined for tibialis anterior and gastrocnemius muscles [26, 32].

Microglia marker levels were not significantly lower in exercised animals compared to non-exercised controls ((*N* = 2; cf = 6), pooled SMD: − 1.62 (95%CI: − 3.70, 0.45)), whereas astrocyte marker levels were significantly higher ((*N* = 2; cf = 6), pooled SMD: 7.50 (95%CI: 3.33, 11.68)) [26, 32].

#### Macrophages

##### Nerve

In the sciatic nerve, the number of macrophages (F4/80) was not significantly lower ((*N* = 2; cf = 2), pooled SMD: − 0.38 (95%CI: − 1.85, 1.09)) in exercised animals compared to control animals [48, 49].

#### Neurotrophins

##### Brainstem

In the brainstem, there was no significant difference in brain-derived neurotrophic factor (BDNF) levels between the control and exercise group ((*N* = 2; cf = 4), pooled SMD: 0.49 (95%CI: − 0.13, 1.11)) [51, 58].

##### Spinal cord

In the spinal cord (unspecified location) there were significantly lower levels of BDNF in the exercise group compared to the control group ((*N* = 2; cf = 4), pooled SMD: − 0.99 (95%CI: − 1.69, − 0.29)) [49, 58].

##### Dorsal root ganglion

In the dorsal root ganglion, BDNF/BDNF mRNA levels ((*N* = 2; cf = 2), pooled SMD: − 1.01 (95%CI: − 1.70, − 0.31)) [24, 44] and nerve growth factor (NGF)/NGF mRNA levels ((*N* = 3; cf = 4), pooled SMD: -0.64 (95%CI: − 1.29, − 0.00)) [24, 40, 44] were significantly reduced in exercised animals. Glial cell line-derived neurotrophic factor (GDNF)/GDNF mRNA levels ((*N* = 2; cf = 2), pooled SMD: − 1.30 (95%CI: − 3.30, 0.69)) were not significantly lower compared to control animals [24, 44].

##### Nerve

BDNF/BDNF mRNA levels were measured in the sciatic [38] or median nerve [33, 59]. There was no significant difference in BDNF/BDNF mRNA levels ((*N* = 3; cf = 3), pooled SMD: 0.21 (95%CI: − 1.38, 1.81)) [33, 38, 59] in exercised compared to non-exercised animals. Insulin-like growth factor-1 (IGF-1) levels measured in the sciatic [50] or median nerve [59] were significantly increased ((*N* = 2; cf = 2), pooled SMD: 1.72 (95%CI: 0.87, 2.57)) in exercised animals compared to non-exercised controls [50, 59].

##### Serum

Serum levels of IGF-1 were not significantly increased ((*N* = 2; cf = 2), pooled SMD: 3.35 (95%CI: − 2.59, 9.28)) in exercised animals compared to controls [35, 59].

##### Muscle

BDNF levels measured in the forearm extrinsic finger flexor muscles [59] or soleus muscle [52] were significantly increased ((*N* = 2; cf = 3), pooled SMD: 3.12 (95%CI: 2.00, 4.24)) in exercised animals. IGF-1/IGF-1 mRNA levels in the red muscle of the gastrocnemius [35], triceps surae [50] and forearm extrinsic finger flexor muscles [59] were not significantly altered ((*N* = 3; cf = 3), pooled SMD: 0.97 (95%CI: − 1.27, 3.22)) in exercised animals in comparison with the control group.

#### Cytokines

##### Brainstem

Levels of interleukin (IL)-1β in the brainstem did not differ significantly ((*N* = 2; cf = 3), pooled SMD: 0.24 (95%CI: − 1.43, 1.91)) between exercised and control animals [55, 58].

##### Spinal cord

In the spinal cord (unspecified location), IL-1β levels ((*N* = 2; cf = 3), pooled SMD: − 0.54 (95%CI: − 1.18, 0.10)) [54, 58] and IL-10 levels ((*N* = 2; cf = 3), pooled SMD: 0.60 (95%CI: − 0.39 1.60)) [36, 54] were not significantly different between exercised animals compared to control animals.

##### Nerve

In the sciatic nerve, tumor necrosis factor-α (TNF-α) levels were significantly reduced ((*N* = 5; cf = 10), pooled SMD: − 1.10 (95%CI: − 1.81, − 0.39)) [28, 29, 47, 50, 54], while IL-1β levels were not significantly lower ((*N* = 3; cf = 4), pooled SMD: − 0.89 (95%CI: − 1.98, 0.21)) [29, 50, 54] in exercised animals compared to controls. Levels of IL-6 ((*N* = 4; cf = 8), pooled SMD: − 0.42 (95%CI: − 1.78, 0.93)) [28, 46, 47, 49] and IL-6 receptor ((*N* = 2; cf = 2), pooled SMD: 1.36 (95%CI: − 3.16, 5.88)) [46, 54] in the sciatic nerve were not significantly different from controls.

Levels of IL-1 receptor antagonist (IL-1RA) ((*N* = 2; cf = 2), pooled SMD: 0.64 (95%CI: − 0.70, 1.99)) [49, 50], IL-4 ((*N* = 2; cf = 2), pooled SMD: 0.54 (95%CI: − 0.62, 1.69)) [49, 50] and IL-10 ((*N* = 3; cf = 7), pooled SMD: 0.40 (95%CI: − 0.54, 1.35)) [28, 47, 54] in the sciatic nerve were not significantly higher in exercised animals compared to control animals.

##### Muscle

The levels of TNF-α/TNF-α mRNA measured in the triceps surae [50] or red muscle of the gastrocnemius [35] were significantly reduced ((*N* = 2; cf = 2), pooled SMD: − 1.06 (95%CI: − 2.12, − 0.01)) [35, 50] in exercised animals compared to controls. Levels of IL-1β/ IL-1β mRNA in the triceps surae [50] or tibialis anterior muscle [35] of exercised animals did not significantly differ from control animals ((*N* = 2; cf = 2), pooled SMD: − 0.57 (95%CI: − 1.52, 0.39)) [35, 50].

#### Neurotransmitters

##### Brainstem

Serotonergic 5-HT2A receptor/5-HT2A receptor mRNA levels ((*N* = 2; cf = 2), pooled SMD: 1.25 (95%CI: 0.49, 2.01)) were significantly higher in exercised animals than in control animals [51, 55].

##### Ventral horn

Motoneuron levels of glutamic acid decarboxylase 67 (GAD67) were not significantly higher in exercised animals compared to controls ((*N* = 2; cf = 5), pooled SMD: 0.88 (95%CI: -0.38, 2.13)) [53, 57].

#### Synaptic stripping

##### Ventral horn

Motoneuron levels of synaptophysin ((*N* = 3; cf = 10), pooled SMD: 2.05 (95%CI: 1.32, 2.77)) [21, 26, 32] and vesicular glutamate transporter 1 (VGlut1) ((*N* = 5; cf = 15), pooled SMD: 1.38 (95%CI: 0.62, 2.15)) [21, 26, 32, 53, 57] were significantly higher in exercised animals, while perineuronal nets (PNNs) ((*N* = 3; cf = 10), pooled SMD: 0.39 (95%CI: − 0.31, 1.09)) [21, 26, 32] were not significantly different compared to controls. Motoneuron levels of vesicular GABA transporter 1 (VGat1) ((*N* = 2; cf = 6), pooled SMD: − 2.99 (95%CI: − 4.72, − 1.26)) were significantly lower in exercised animals compared to control animals [26, 32].

#### Other neuroimmune substances

##### Nerve

Levels of Growth Associated Protein 43 (GAP43) measured in the sciatic [37] or median [33] nerve ((*N* = 2; cf = 3), pooled SMD: 0.76 (95%CI: 0.17, 1.35)) were significantly elevated in exercised animals compared to controls.

#### Post hoc sensitivity analysis

Because one study [35] reported exactly the same but opposite IGF-1 mRNA levels in two muscles (i.e., tibialis anterior and the red muscle of the gastrocnemius), a post hoc sensitivity analysis was performed. Sensitivity analyses using the results for the tibialis anterior muscle instead of the red muscle of the gastrocnemius, showed a significant increase of IGF-1/IGF-1 mRNA ((*N* = 3; cf = 3), pooled SMD: 1.77 (95%CI: 1.03, 2.52)). The most conservative effect estimate (i.e., red muscle of the gastrocnemius) has been retained.

#### Subgroup analyses and publication bias

It was not possible to perform subgroup analyses or assess publication bias, because insufficient studies were available. The risk of bias of all studies was unclear.

## Discussion

The aim of this systematic review and meta-analyses was to determine the effect of aerobic exercise on neuroimmune responses in animals with a traumatic peripheral neuropathy. Results from 40 studies were included. In general, the findings indicate that aerobic exercise has a positive influence on neuroimmune responses that occur following traumatic peripheral neuropathy. These positive effects are seen at local and remote locations relative to the lesion site.

### Effects of aerobic exercise

#### Nerve

In the early stages after peripheral nerve injury, macrophages are involved in the increased release of pro-inflammatory cytokines in the nerve [7, 12, 60]. Mainly macrophages located in the DRG were found to be responsible for the development and maintenance of hypersensitivity after peripheral neuropathy [4]. Downregulation of pro-inflammatory cytokines by an increased differentiation into anti-inflammatory M2 macrophages under the influence of IL-4 is suggested to play a role in reducing hypersensitivity after peripheral nerve injury [4, 7]. One of the proposed effects of exercise lies in macrophage phenotype polarization at the site of injury from a pro-inflammatory (M1) to an anti-inflammatory (M2) state [60]. Based on our findings however, no definite conclusions on the influence of aerobic exercise on macrophage levels and phenotype polarization at or around the site of injury could be drawn from two studies [48, 49] with a limited number of animals, indicating more research on this subject is warranted.

Pro-inflammatory cytokines, such as TNF-α, IL-1β and IL-6, are released in response to injury [61]. Increased levels of pro-inflammatory cytokines incite further proliferation of these cytokines, causing an escalation of pro-inflammatory processes [61]. Anti-inflammatory cytokines, such as IL-10 and IL-4, keep the inflammatory response in check by downregulating pro-inflammatory processes [61]. Although a variety of pro- and anti-inflammatory cytokine levels in the nerves have been investigated in response to aerobic exercise, the only significant difference was found for a decreased level of TNF-α in the sciatic nerve [28, 29, 47, 50, 54]. Given that TNF-α is considered a key mediator of neuropathic pain [61], lower levels of TNF-α imply an anti-inflammatory influence of aerobic exercise. Additionally, although not significant, a general trend of lower levels of pro-inflammatory cytokines and higher levels of anti-inflammatory cytokines could be observed in exercised animals, compared to control animals. However, additional research is needed to draw more definite conclusions.

GAP43 is involved in axonal growth and is considered an indicator for rapid neuron regeneration [62, 63]. Evidence suggests that in the early stages after dorsal root injury, mainly GAP43 immunoreactive neurons support axonal growth, followed later by neurons that are not GAP43 immunoreactive [62]. The increased levels of GAP43 in the nerve of exercised animals [33, 37], measured at 1–3 weeks after nerve injury, therefore suggest an improved nerve regeneration in trained animals. Physical activity induced BDNF at the dorsal horn level has been implicated to play a role in the enhanced GAP43 expression and subsequent increased neuroplasticity [64, 65]. In this review however, BDNF/BDNF mRNA levels in the nerve of exercised animals were not significantly different from control animals [33, 38, 59]. These results might be explained by the difference in timing of outcome measurement and the low number of animals included in the studies. Additional research is needed to shed light on the impact aerobic exercise has on BDNF levels at the site of injury. The significant increase of insulin growth factor-1 (IGF-1) levels in the nerve found in our study [50, 59] also supports the notion that exercise leads to enhanced nerve regeneration [50, 61].

#### Dorsal root ganglion

Neurotrophin levels in the DRG were lower in exercised animals, with a significant reduction in BDNF/BDNF mRNA [24, 44] and NGF/NGF mRNA levels [24, 40, 44] and a non-significant reduction of GDNF/GDNF mRNA levels [24, 44]. Given that upregulation of BDNF and NGF in the dorsal horn after nerve injury is associated with an enhanced pro-inflammatory response and hyperalgesia, these findings are indicative of a normalization of neurotrophic levels in response to exercise, resulting in an anti-inflammatory response [14, 49, 61]. Unfortunately, no other meta-analyses could be performed at the DRG level.

#### Spinal cord

Significantly lower spinal cord BDNF levels were found in physically active animals, compared to controls [49, 58]. Activated microglia in the spinal cord release BDNF, among other molecules, in response to nerve injury [61]. Increased levels of BDNF impact GABA-mediated neuronal inhibitory processes, thus facilitating nociception [11, 61]. The lower BDNF levels found in exercised animals therefore imply a beneficial effect of aerobic exercise. Additionally, although not significant, the reduction of IL-1β and increased levels of IL-10 are also indicative of an anti-inflammatory effect of exercise at the spinal cord level.

After nerve injury, glial cells in the dorsal horn are upregulated, leading to stimulation of first and second order neurons through the release of pro-inflammatory mediators [12, 61]. In the long term, this process can cause plastic changes at the dorsal horn and central sensitization [12]. Microglia change from a homeostatic phenotype into classical activation promptly after peripheral nerve injury, causing neuronal hyperexcitability through the release of pro-inflammatory mediators [11, 12, 60, 61, 66, 67]. The results in our study showed a significant decrease of microglia markers (Iba1 or CD^−^11b^+^) in the dorsal horn [30, 36, 40, 44, 49, 56]. Astrocyte activation occurs later than microglial activation, and is associated with the persistence of neuropathic pain through the release of pro-inflammatory mediators [11, 61, 66]. Inversely, a possible anti-nociceptive influence of astrocytes is proposed through the release of the primarily anti-nociceptive neurotrophin GDNF [61]. The results found in our study showed an overall decrease in levels of astrocyte marker GFAP after exercise [30, 49, 56]. While the GFAP levels measured 7 days post-injury appear to be similar for both groups, a more apparent reduction was seen at later stages (i.e., at 2, 3 and 5 weeks post-injury), indicating that the relative decrease of GFAP levels in physically active animals occurs mainly at a later stage. Overall, these results suggest a positive influence of aerobic exercise on glial cell levels at the dorsal horn. Given that no other meta-analyses could be performed for neuroimmune processes at the dorsal horn, despite the important role the dorsal horn plays in nociceptive pathways, future research should focus on further unravelling the influence of exercise on neuroimmune processes taking place at the dorsal horn.

Axotomy of spinal motoneuron in the ventral horn leads to large-scale synaptic stripping [21, 26, 68]. Microglia have been implicated as an important contributor to synaptic stripping [68]. However, a recent systematic review suggested a paradigm shift away from the notion that microglia are considered the ‘universal synaptic strippers’ [68]. They describe two different forms of synaptic plasticity: a mechanism of synaptic stripping that is influenced, among others, by local microglia, astrocytes and neurotrophin levels. Additionally, these authors propose a process that is microglia-dependent [68]. The levels of microglia marker Iba1 in the ventral horn of exercised animals found in our review were not significantly altered compared to controls [26, 32]. However, significantly higher levels of astrocyte marker GFAP were found in the ventral horn of exercised animals compared to controls [26, 32]. Findings from a recent study imply a rapid astrocyte activation at the ventral horn after peripheral nerve injury, that coincides with increased neurotrophin levels and appears to be beneficial for nerve regeneration and motor function [69]. This may imply that the increased GFAP levels found in exercised animals are associated with a beneficial effect on nerve regeneration. However, the precise role that astrocytes play and the mechanisms associated with higher GFAP levels found in exercised animals compared to controls require further investigation. Several outcomes related to synaptic stripping were examined, showing significantly higher levels of synaptophysin [21, 26, 32] and Vglut1 [21, 26, 32, 53, 57] and significantly lower levels of VGat1 [26, 32] in active animals, compared to controls. PNN levels were not significantly different between groups. These results suggest a beneficial role of physical activity in the reduction of synaptic stripping in motoneurons after peripheral nerve injury. GABA-related GAD67 levels were not significantly altered in exercised animals compared to controls [53, 57].

#### Brain

Three meta-analyses could be performed for neuroimmune responses in the brainstem, while no meta-analyses could be performed for the cerebral cortex, highlighting the need for additional studies in these areas. In the brainstem, we found a significant increase of the serotonin 5-HT2A receptor/5-HT2A receptor mRNA expression [51, 55]. Enhanced serotonergic neurotransmission is one of the proposed mechanisms behind exercise-induced analgesia by facilitating descending inhibitory processes at the dorsal horn [51, 55]. The increased levels of 5-HT receptor levels found in our study are therefore suggestive of a beneficial influence of exercise on serotonergic neurotransmission. No significant difference was found in BDNF [51, 58] and IL-1β [55, 58] levels in the brainstem.

#### Blood/serum

One meta-analysis could be performed for neuroimmune responses in blood or serum, showing a non-significant increase in IGF-1 [35, 59].

#### Muscle

In contrast with results found in the spinal cord and dorsal root ganglion, BDNF levels were significantly higher in the muscles of exercised animals [52, 59]. The increased levels of BDNF found in muscles after aerobic exercise are considered an important part of the muscle reinnervation process that takes place after nerve injury [52]. This suggests that the higher BDNF levels as a result of physical activity are beneficial for recovery [52].

The significantly reduced levels of the pro-inflammatory cytokine TNF-α found in exercised animals compared to controls [35, 50] are indicative of an anti-inflammatory effect of aerobic exercise. Although not significant, the observed decrease in pro-inflammatory IL-1β [35, 50] and higher levels of IGF-1 [35, 50, 59] also suggests anti-inflammatory responses in muscles of exercised animals. However, more studies are needed to confirm these findings.

### Limitations and recommendations

Several limitations should be considered when interpreting the results reported in this systematic review and meta-analyses. A wide range of neuroimmune responses was measured in the included studies. However, the number of studies that measured these outcomes was generally low, making it impossible to perform meta-analyses for a large proportion of the neuroimmune responses. Moreover, most studies included only small numbers of animals (e.g., 4–5 animals per group), which tends to make effect measures imprecise.

There was a large degree of heterogeneity between the included studies. A variety of peripheral neuropathy models and animal species and strains were used in the studies. Although all intervention groups consisted of aerobic exercise, the exercise type (e.g., treadmill training, swimming, voluntary wheel running), duration, frequency and intensity differed substantially. Considering the large variability in timing, length and intensity of the exercise programs, subgroup analyses were not possible. Additionally, the timepoints at which the outcomes were measured varied considerably leading to inconsistency. Considering that different phases of recovery following peripheral neuropathy require a different balance of pro- and anti-inflammatory processes, the optimal levels of pro- and anti-inflammatory substances differ over time. To account for the heterogeneity in the study design, random effect models were used to estimate effects.

The focus of this review was on the neuroimmune processes that occur after aerobic exercise, therefore no functional outcomes were included. It would be informative to gain insight in the relations between neuroimmune outcomes and functional outcomes to determine whether changes in neuroimmune processes also lead to better functional outcomes, however this was beyond the scope of this review.

Only 10 out of 40 studies included female animals. In recent years, it has become apparent that (neuro)immune responses to peripheral nerve injury differ between male and female animals [70–72]. Unfortunately, there were too few independent comparisons per outcome measure to perform reliable subgroup analyses for sex. Nevertheless, the observation that there are too few studies conducted using female animals highlights the need for future studies. These studies should investigate the extent to which neuroimmune responses associated with exercise differ between male and female animals after peripheral nerve injury.

Risk of bias assessment showed that most risk of bias criteria were scored as ‘unclear’ in the included studies. This makes it difficult to judge the impact of important sources of bias, such as allocation sequence generation, application and concealment, similarity of the groups at baseline, blinding of caregivers, researchers and outcome assessors and selective reporting, on the results. Future animal studies should adhere to the ARRIVE reporting guidelines for animal studies [73] to ensure more clarity on the methods used and provide a clearer picture of the influence potential sources of bias might have had on the findings.

## Conclusions

Overall, the findings of this systematic review and meta-analyses suggest that aerobic exercise has beneficial effects on neuroimmune responses across various anatomical locations along the neuraxis. Additional research is needed to further elucidate the mechanisms underlying the effect of exercise on neuroimmune processes and/or substances.

## Supplementary Information

**Additional file 1.** Literature search, shows the literature search for MEDLINE (via Pubmed), EMBASE and Web of Science.

**Additional file 2.** Overview meta-analyses, shows the forest plots for all meta-analyses organized per class of neuroimmune outcome per anatomical location.

**Additional file 3.** Overview Non-meta-analyses, shows the forest plots for all Non-meta-analyses organized per class of neuroimmune outcome per anatomical location.

## Data Availability

The datasets generated during and/or analyzed during the current study are available from the corresponding author on reasonable request.

## Abbreviations

DRG: Dorsal root ganglion
PRISMA: Preferred Reporting Items for Systematic reviews and Meta-Analyses
PROSPERO: International Prospective Register of Systematic Reviews
SYRCLE: Systematic Review Center for Laboratory Animal Experimentation
SMD: Standardized mean differences
95%CI: 95% Confidence interval
PSL: Partial sciatic nerve ligation
SNI: Spared nerve injury
SNL: Spinal nerve ligation
GFAP: Glial fibrillary acidic protein
BDNF: Brain-derived neurotrophic factor
NGF: Nerve growth factor
GDNF: Glial cell line-derived neurotrophic factor
IGF-1: Insulin-like growth factor-1
IL: Interleukin
TNF-α: Tumor necrosis factor-α
GAD67: Glutamic acid decarboxylase 67
VGlut1: Vesicular glutamate transporter 1
PNN: Perineuronal net
VGat1: Vesicular GABA transporter 1
GAP43: Growth Associated Protein 43
