# Supplementary material for: The effects of aerobic exercise on neuroimmune responses in animals with traumatic peripheral nerve injury: a systematic review with meta-analyses
Source: J Neuroinflammation. 2023 May 3;20:104. doi: 10.1186/s12974-023-02777-y (PMC10155410; doi:10.1186/s12974-023-02777-y)
Supplement: Supplementary file 2 — Additional file 2. Overview meta-analyses, shows the forest plots for all meta-analyses organized per class of neuroimmune outcome per anatomical location. [file 12974_2023_2777_MOESM2_ESM.docx]

**Additional file 2. Overview Meta analyses**

| **Neuroinflammation markers** |
| --- |
| **DORSAL HORN** |
| **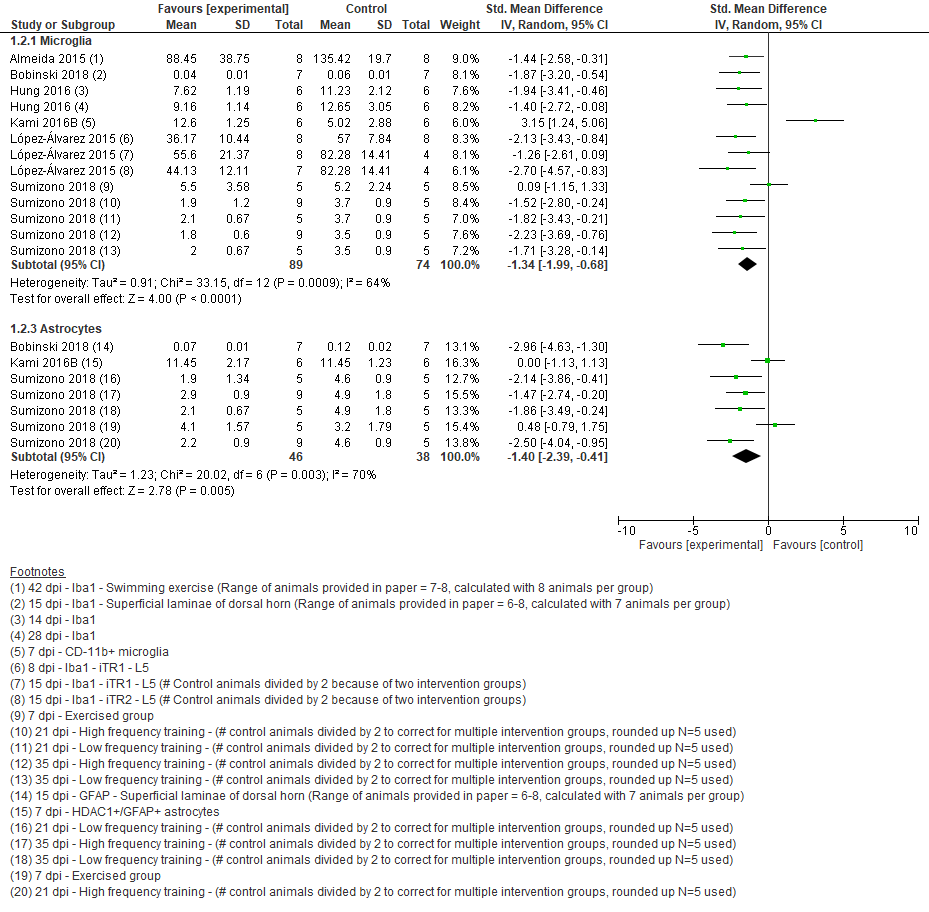** |

| **VENTRAL HORN (Motoneurons)** |
| --- |
| **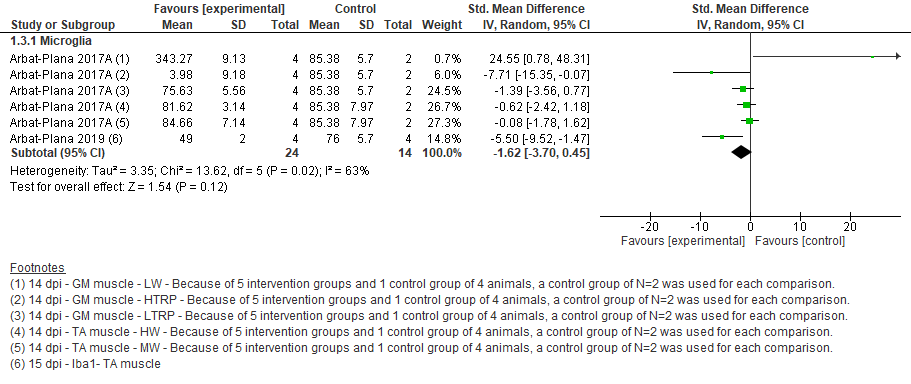**  **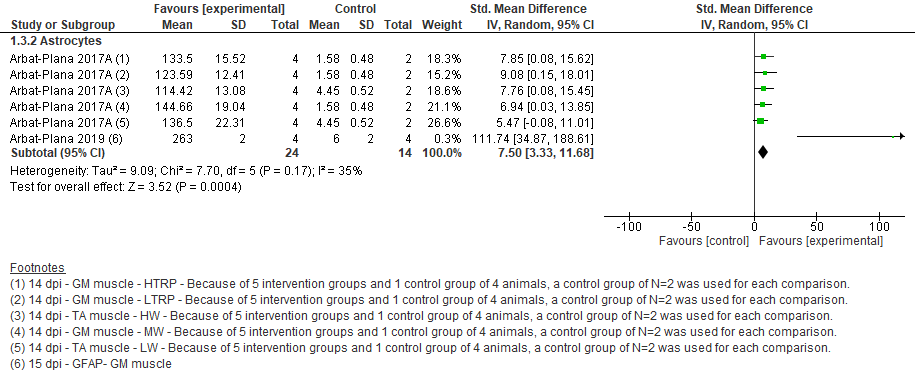** |
| **Macrophages** |
| **NERVE** |
| **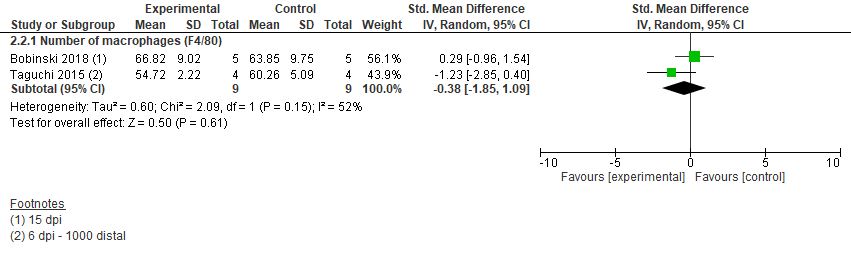** |
| **NEUROTROPHINS** |
| **BRAIN** |

| **BRAINSTEM** |
| --- |
| **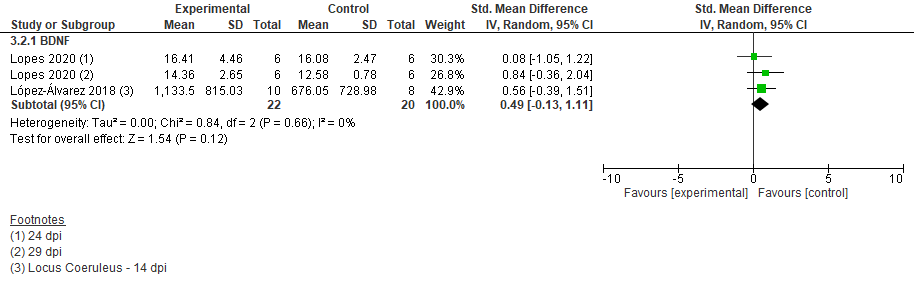** |
| **SPINAL CORD (Unspecified)** |
| **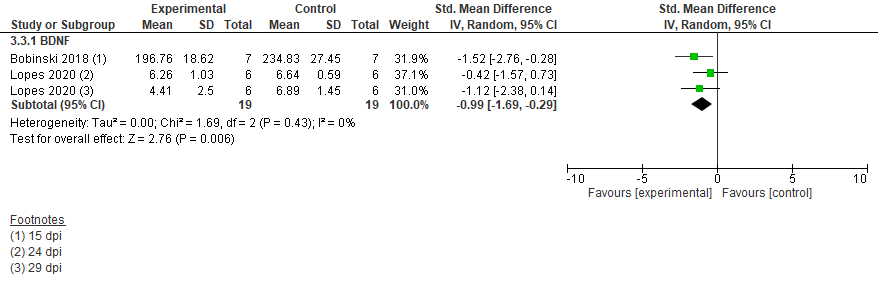** |
| **DORSAL ROOT GANGLION** |
| **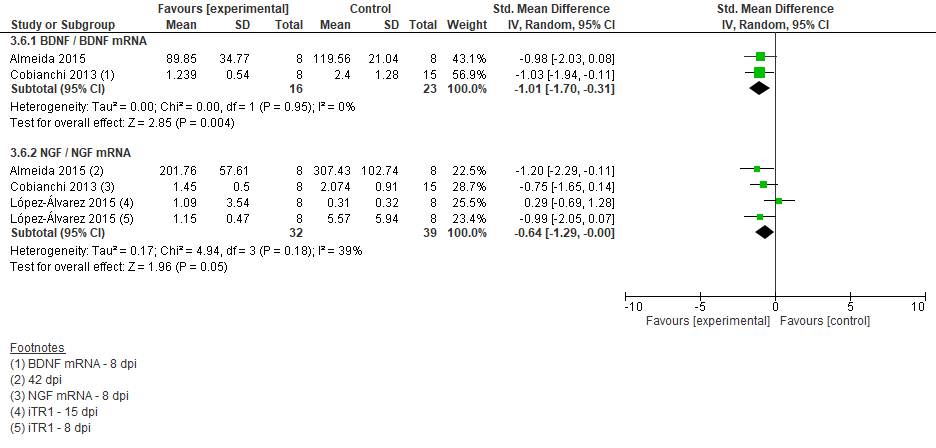**  **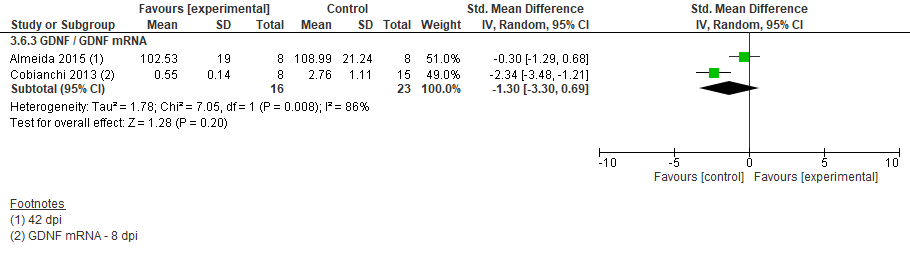** |
| **NERVE** |
| **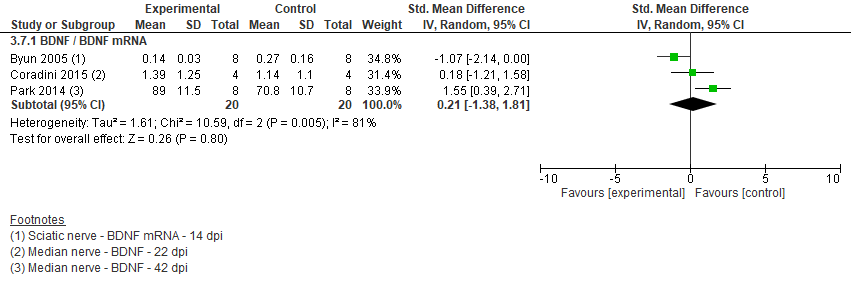**  **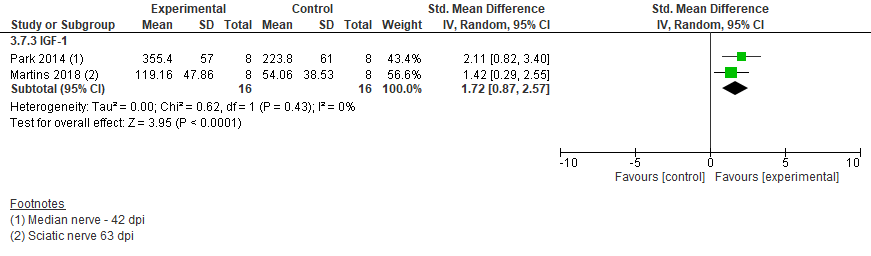** |
| **BLOOD/SERUM** |
| **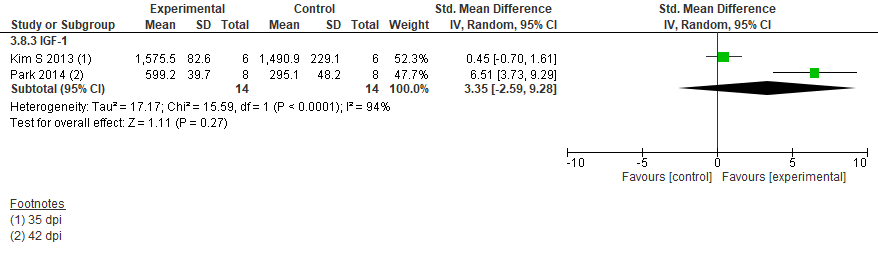** |

| **MUSCLE** |
| --- |
| **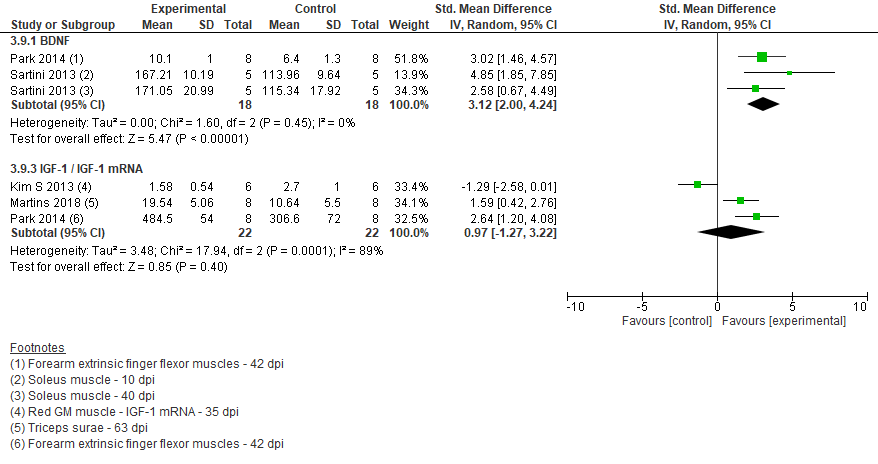** |
| **SENSITIVITY ANALYSES IGF-1 / IGF-1 mRNA** |
| **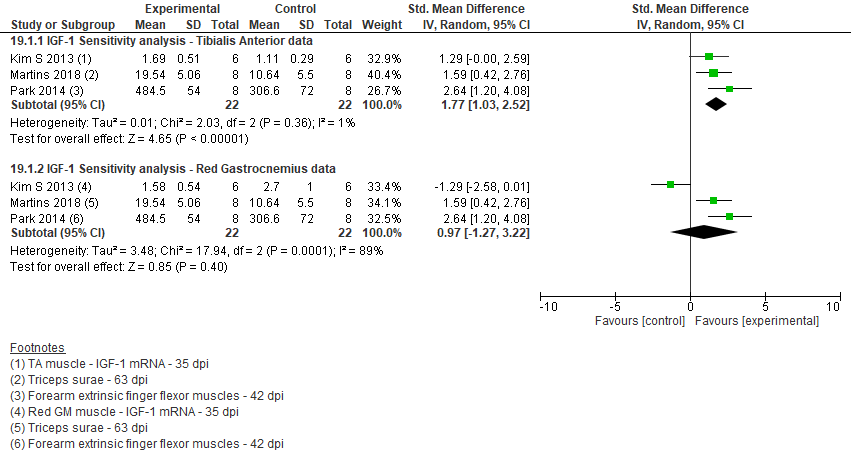** |
| **CYTOKINES** |
| **BRAINSTEM** |
| **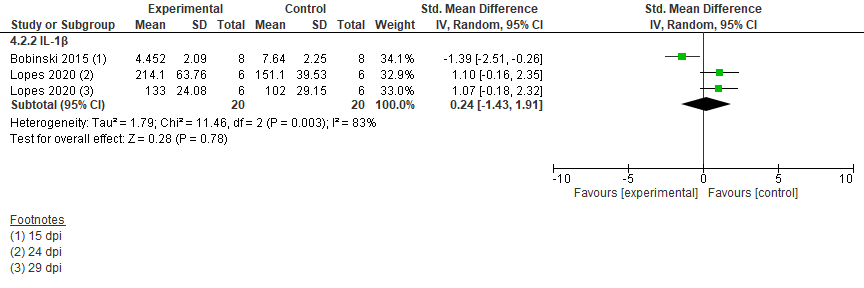** |
| **SPINAL CORD (Unspecified)** |
| **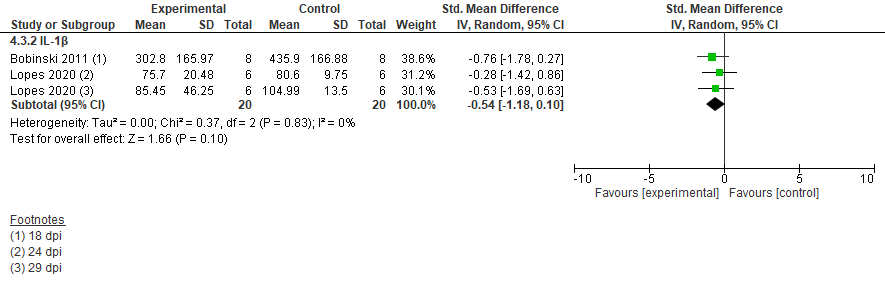**  **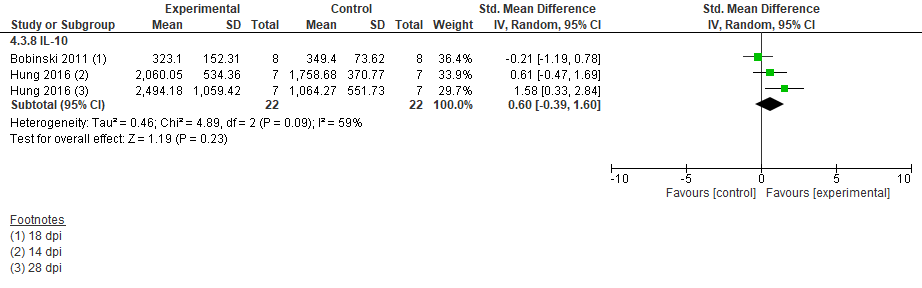** |

| **NERVE** |  |
| --- | --- |
| **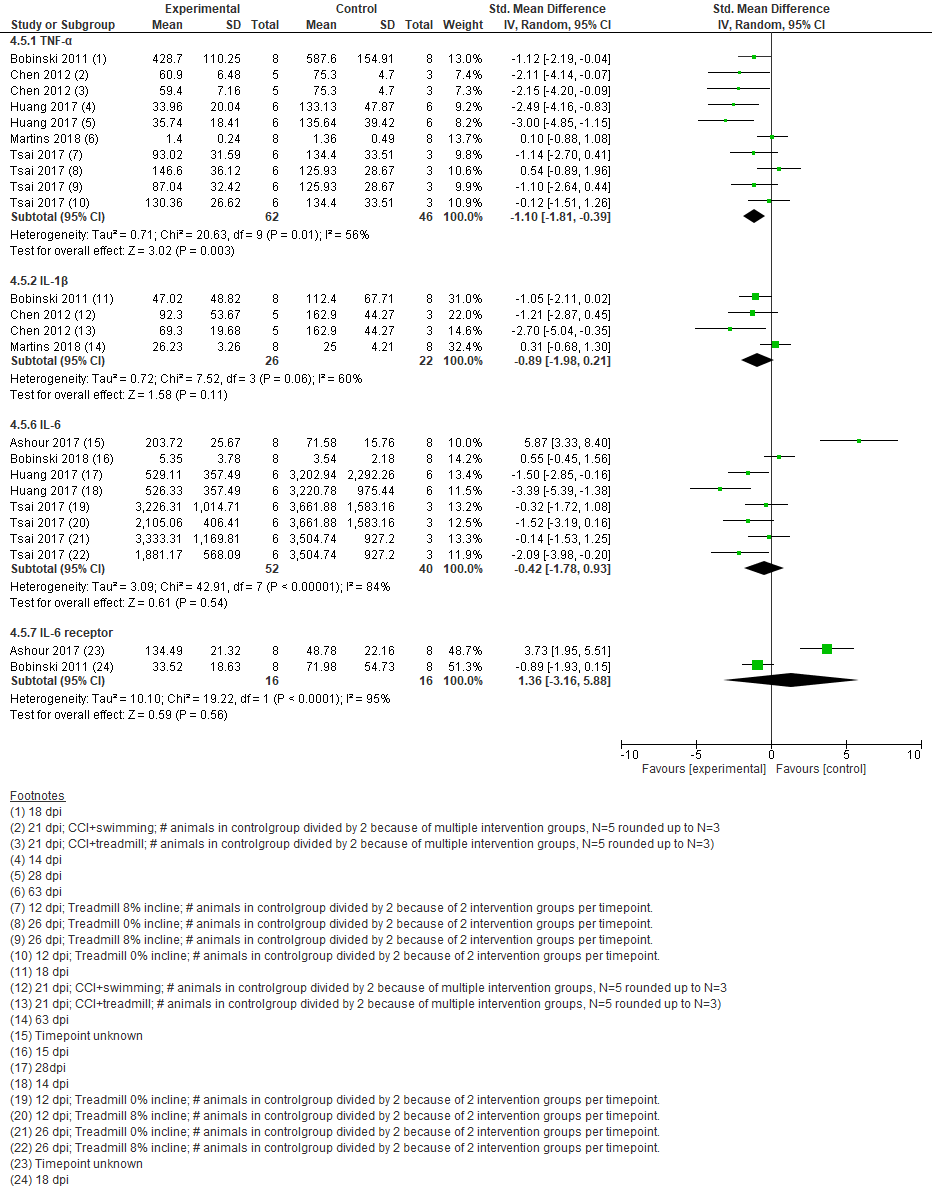**  **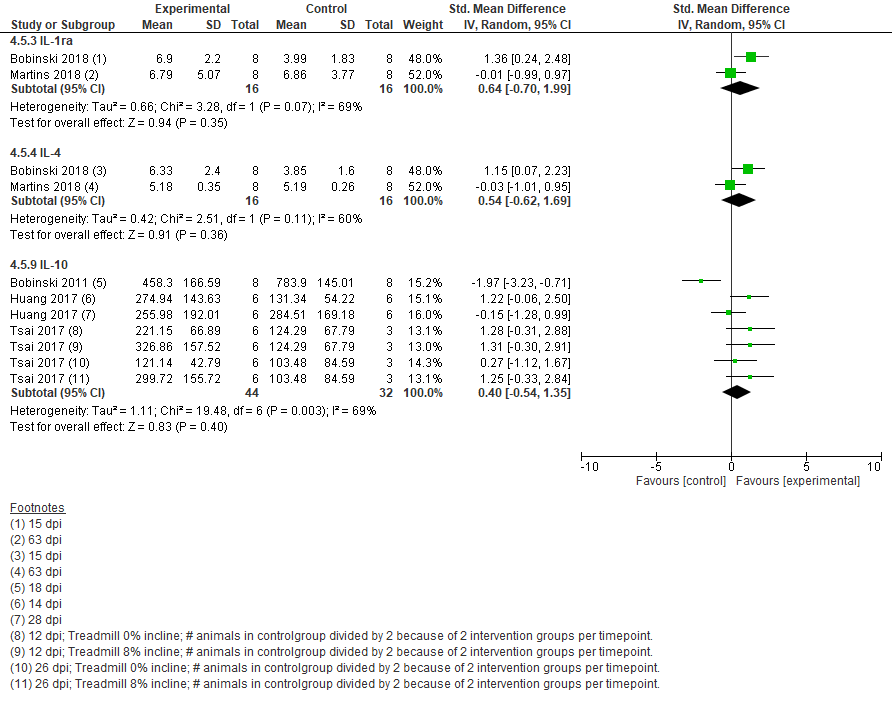** |  |
| **MUSCLE** |  |
| **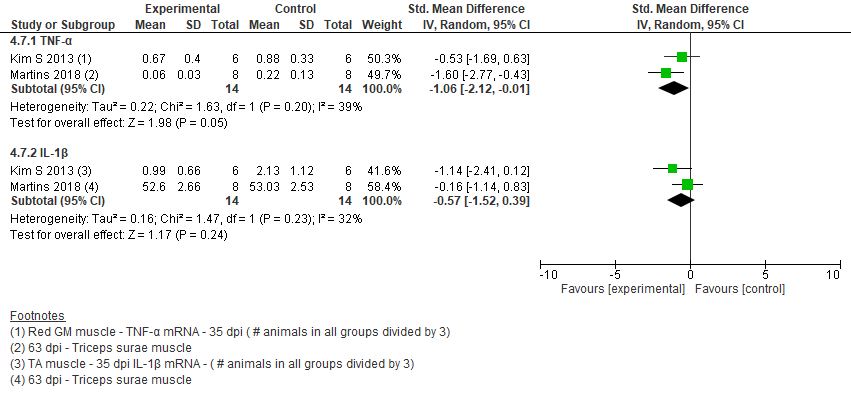** |  |
| **NEUROTRANSMITTERS** |  |
| **Serotonin** |  |
| **BRAINSTEM** |  |
| **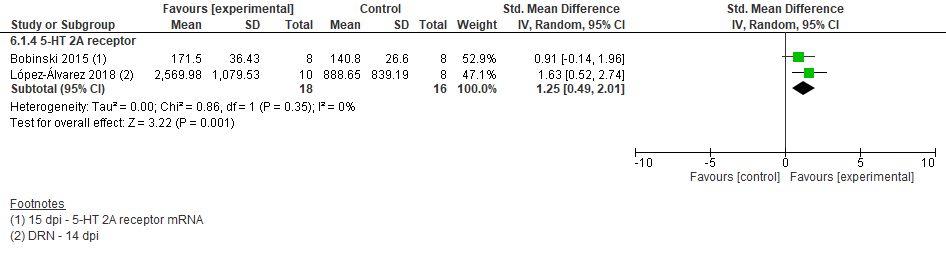** |  |
| **GABA** |  |
| **VENTRAL HORN** |  |
| **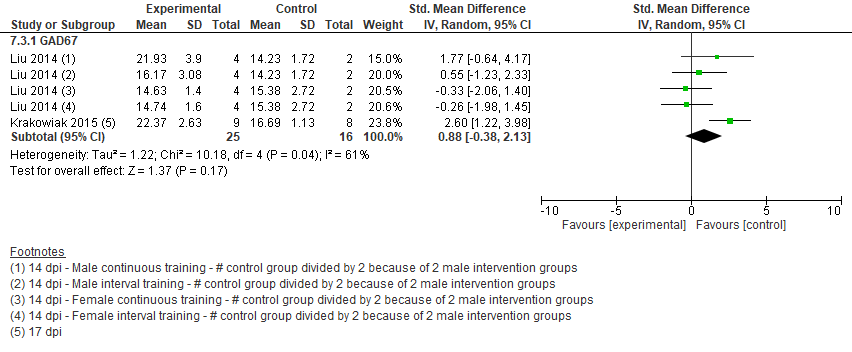** |  |
| **SYNAPTIC STRIPPING** |  |
| **VENTRAL HORN (Motoneurons)** |  |
| **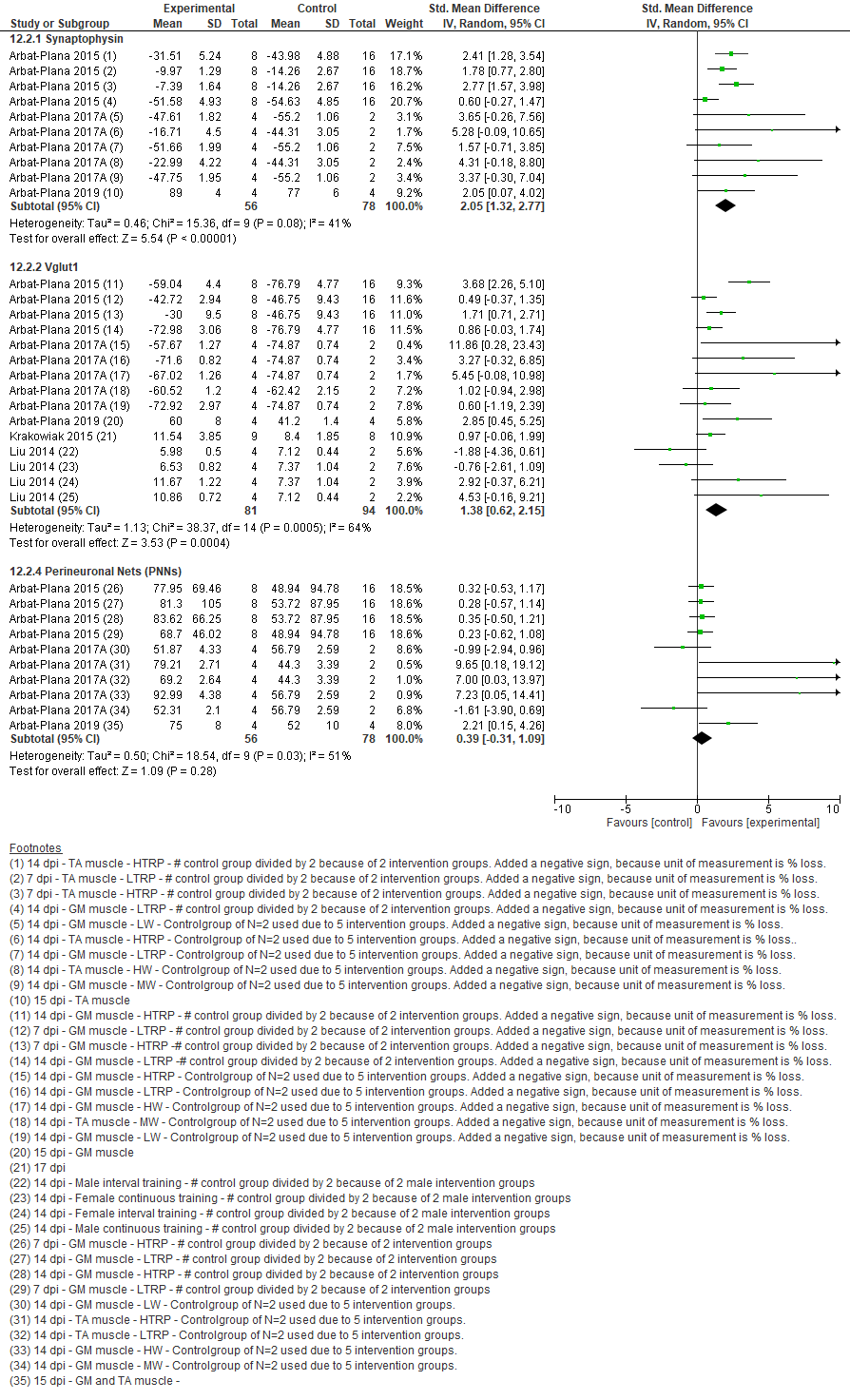**  **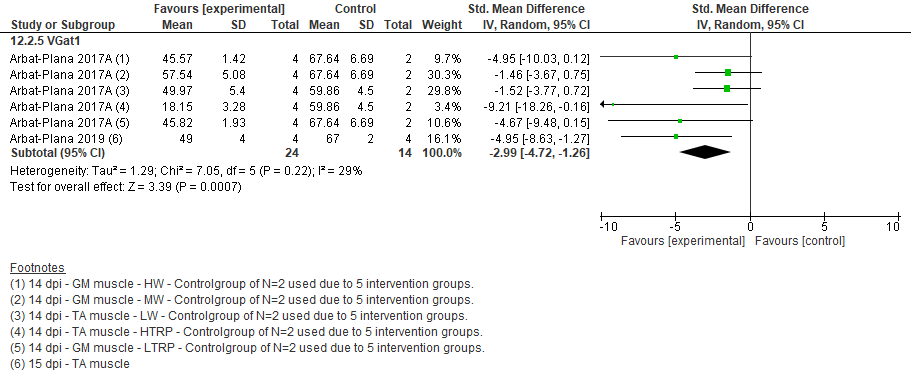** |  |
| **OTHER** | |
| **GAP43** | |
| **NERVE** | |
| **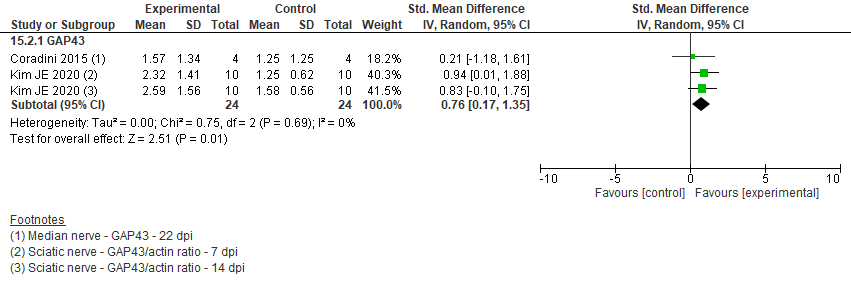** | |
