# Supplementary material for: The effects of aerobic exercise on neuroimmune responses in animals with traumatic peripheral nerve injury: a systematic review with meta-analyses
Source: J Neuroinflammation. 2023 May 3;20:104. doi: 10.1186/s12974-023-02777-y (PMC10155410; doi:10.1186/s12974-023-02777-y)
Supplement: Supplementary file 3 — Additional file 3. Overview Non-meta-analyses, shows the forest plots for all Non-meta-analyses organized per class of neuroimmune outcome per anatomical location. [file 12974_2023_2777_MOESM3_ESM.docx]

**Additional file 3. Overview Non-Meta-Analyses**

| **C1. Neuroinflammation markers** |
| --- |
| **BRAINSTEM** |
| **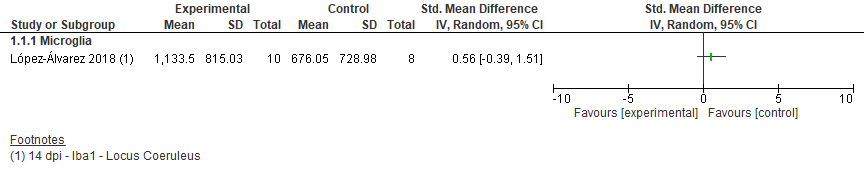** |
| **DORSAL HORN** |
| **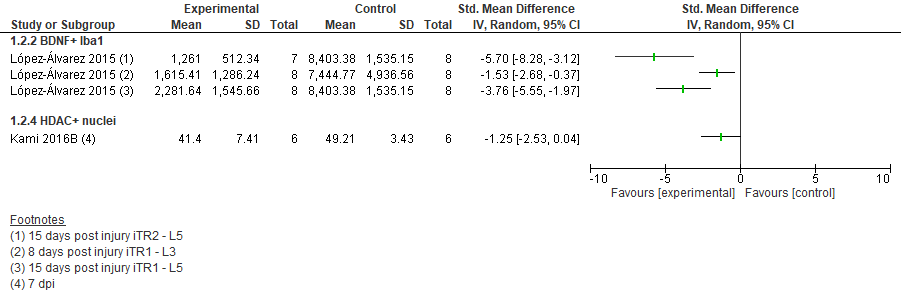** |
| **DORSAL ROOT GANGLION** |
| **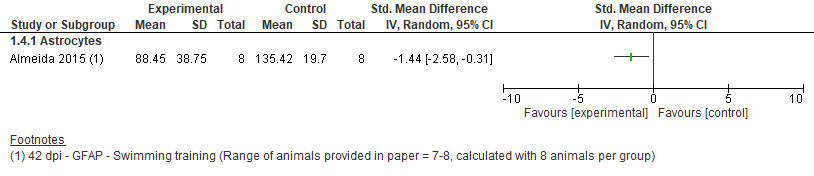** |
| **C2. Macrophages** |
| **SPINAL CORD (Unspecified)** |
| **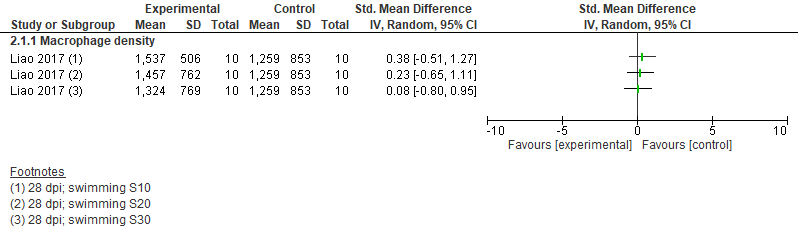** |
| **NERVE** |
| **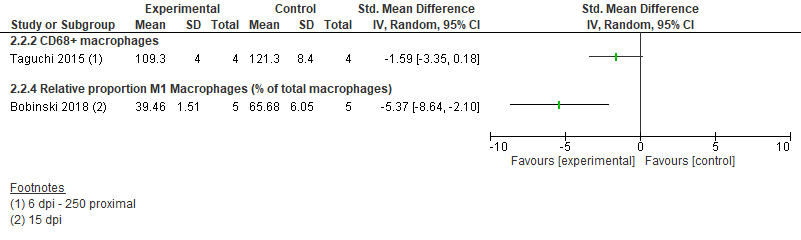**  **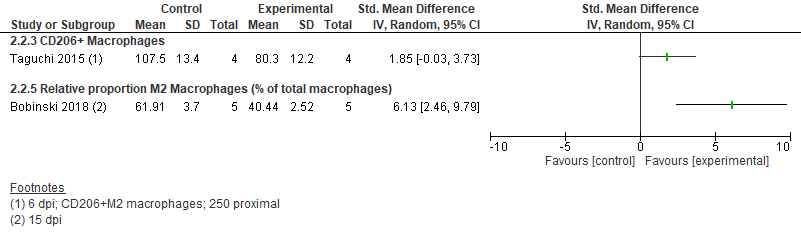** |
| **C3. NEUROTROPHINS** |
| **BRAIN** |
| **Cerebral cortex** |
| **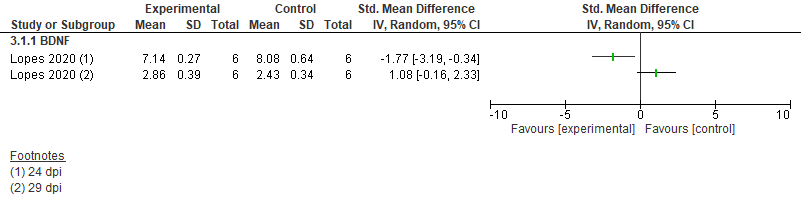** |
| **SPINAL CORD (Unspecified)** |
| **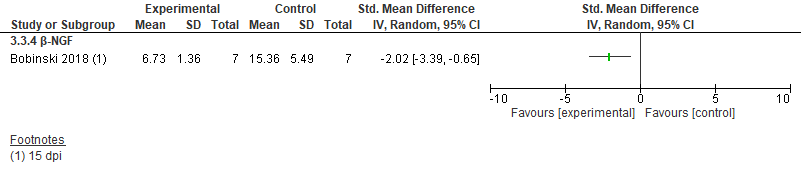** |

| **DORSAL HORN** |
| --- |
| **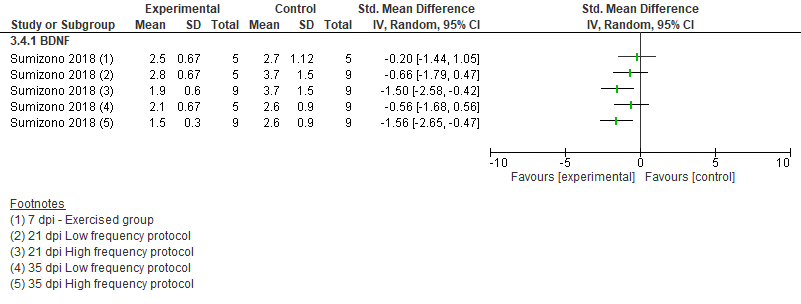**  **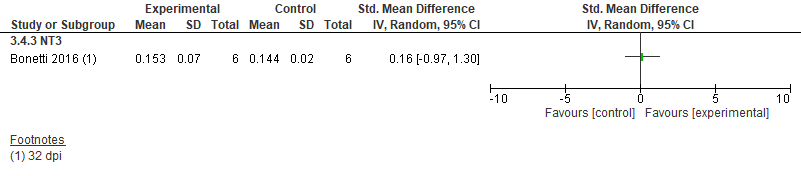** |
| **VENTRAL HORN** |
| **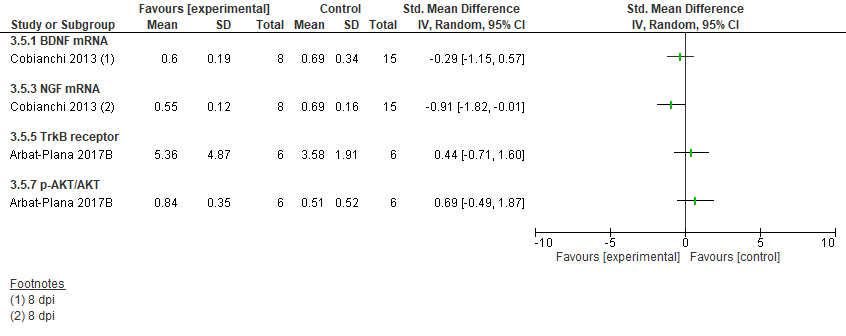**  **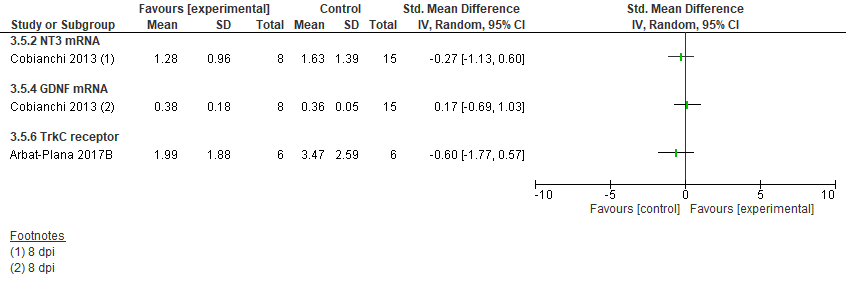** |
| **DORSAL ROOT GANGLION** |
| **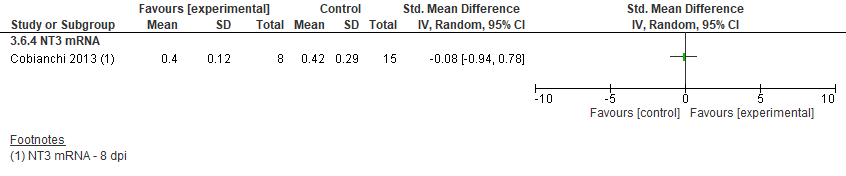** |
| **NERVE** |
| **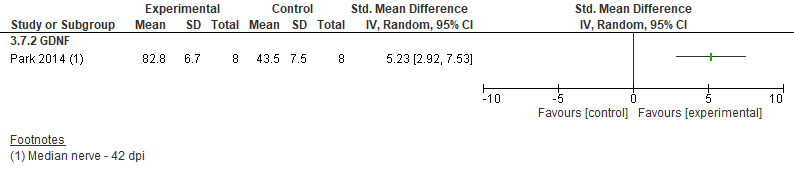** |
| **BLOOD/SERUM** |
| **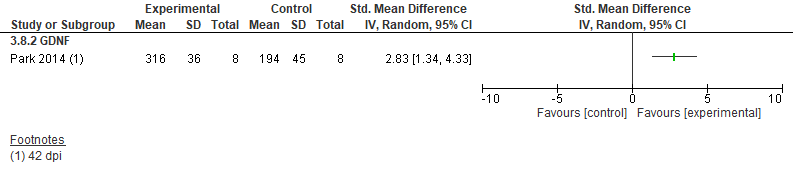** |
| **Muscle** |
| **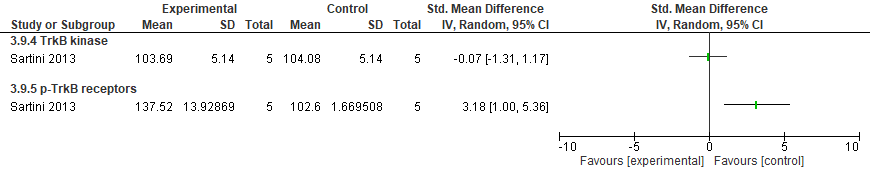**  **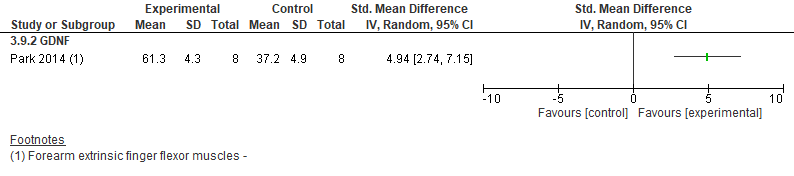** |
| **LIVER** |
| **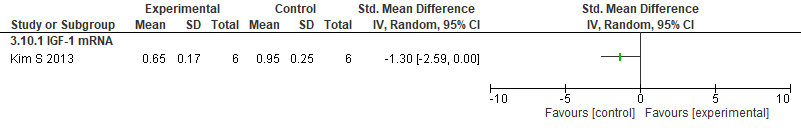** |
| **C4. CYTOKINES** |
| **CEREBRAL CORTEX** |
| **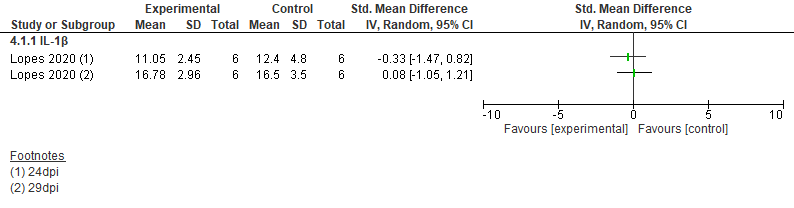**  **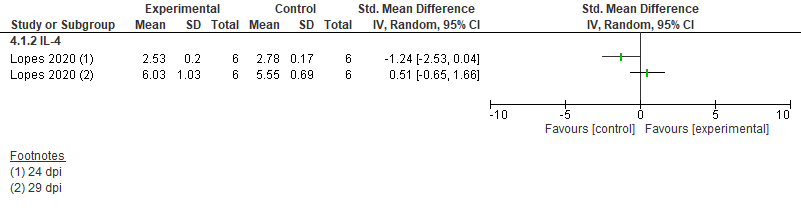** |
| **BRAINSTEM** |
| **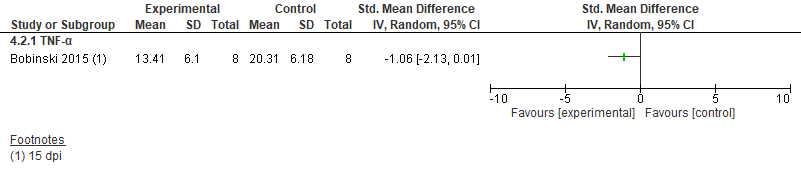**  **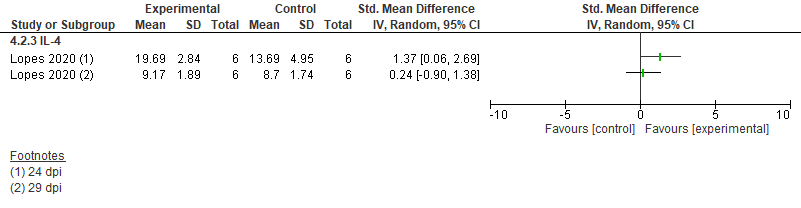** |
| **SPINAL CORD (Unspecified)** |
| **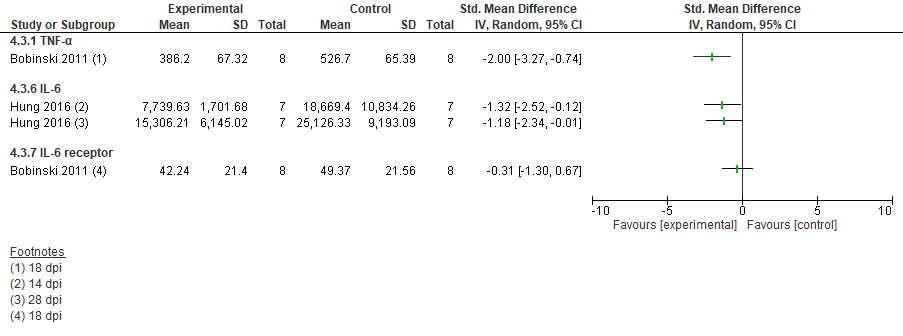**  **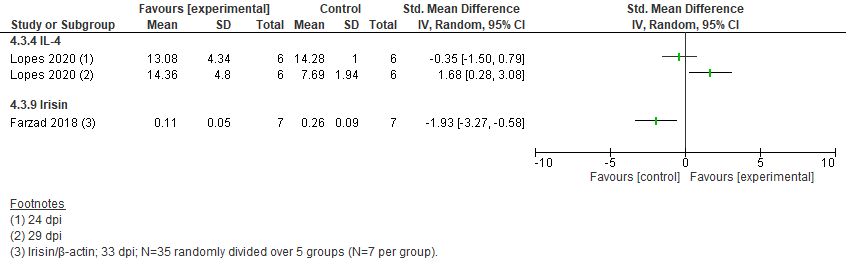** |
| **DORSAL HORN** |
| **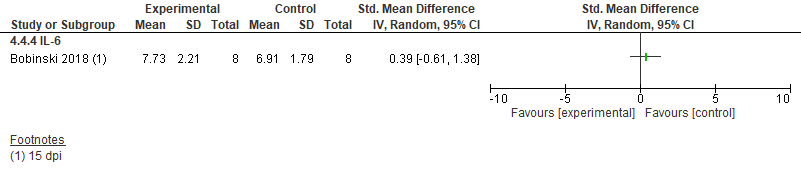**  **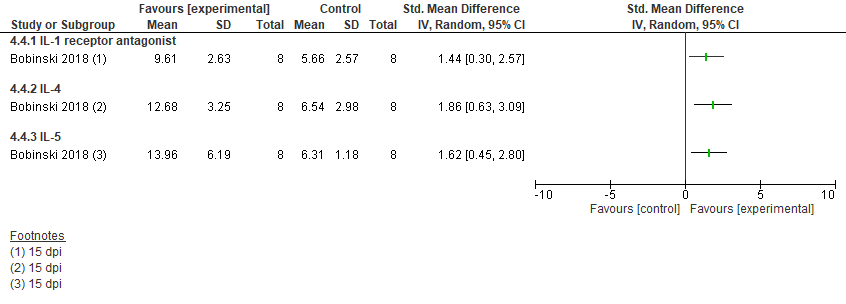** |
| **NERVE** |
| **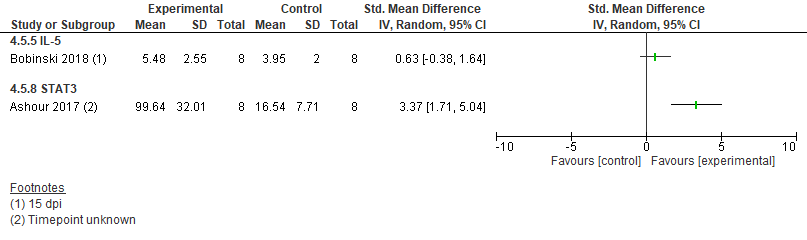** |
| **BLOOD/SERUM** |
| **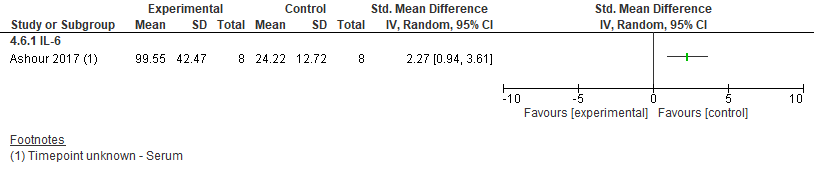** |
| **MUSCLE** |
| **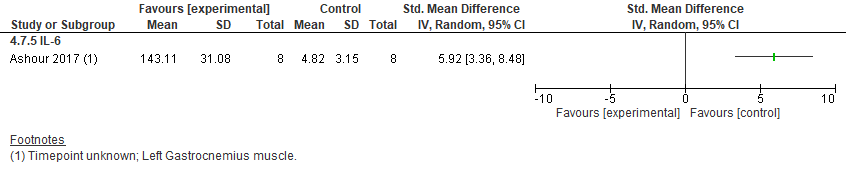**  **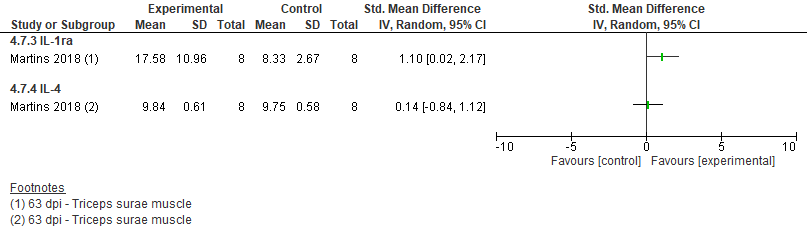** |
| **CEREBROSPINAL FLUID** |
| **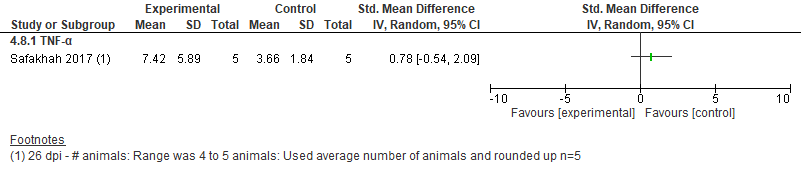** |
| **NEUROTRANSMITTERS** |
| **C5. (Nor)adrenaline** |
| **BRAINSTEM** |
| **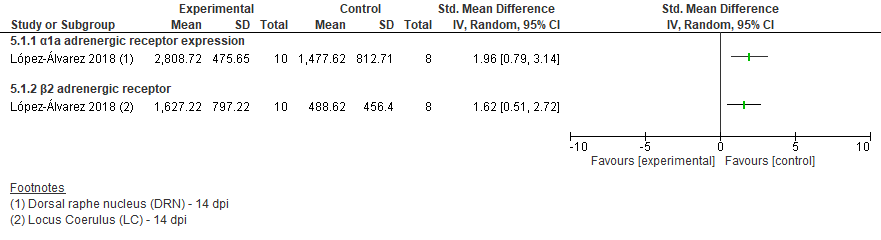** |
| **DORSAL HORN** |
| **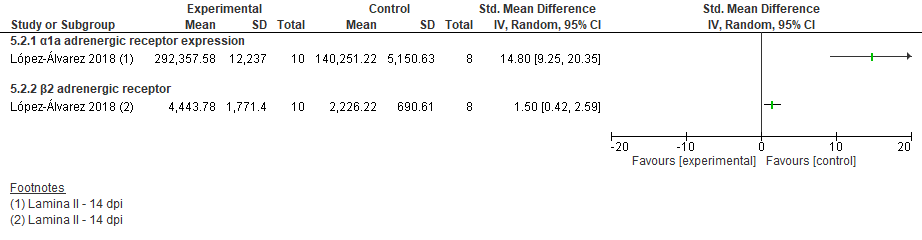** |
| **C6. Serotonin** |
| **BRAINSTEM** |
| **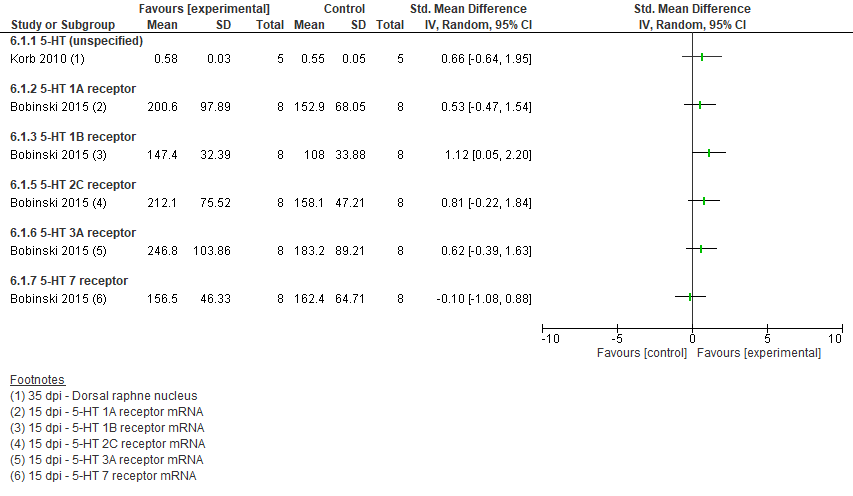**  **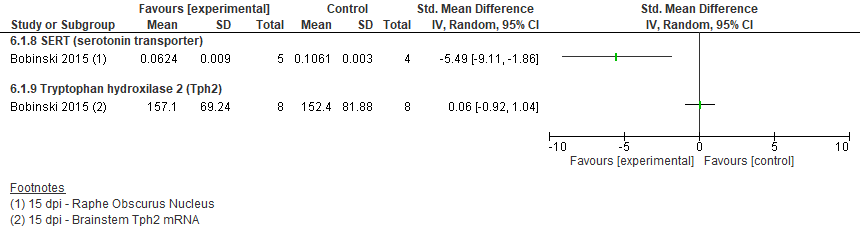** |
| **DORSAL HORN** |
| **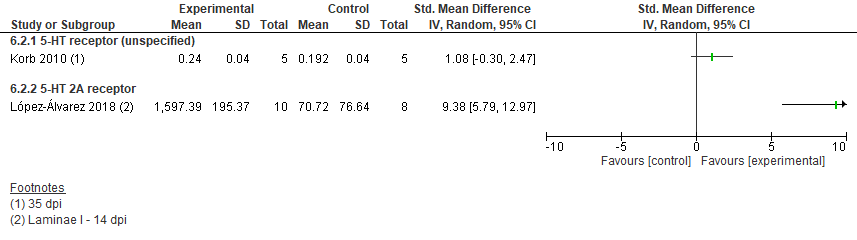** |
| **VENTRAL HORN** |
| **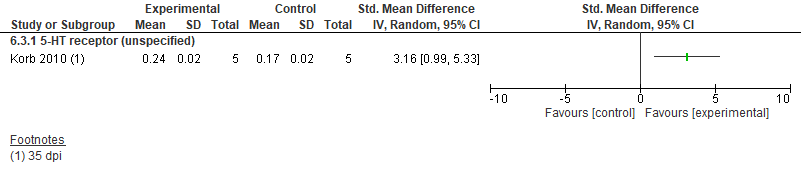** |
| **C7. GABA** |
| **SPINAL CORD (unspecified)** |
| **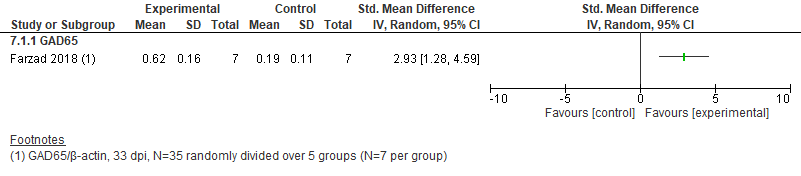** |
| **DORSAL HORN** |
| **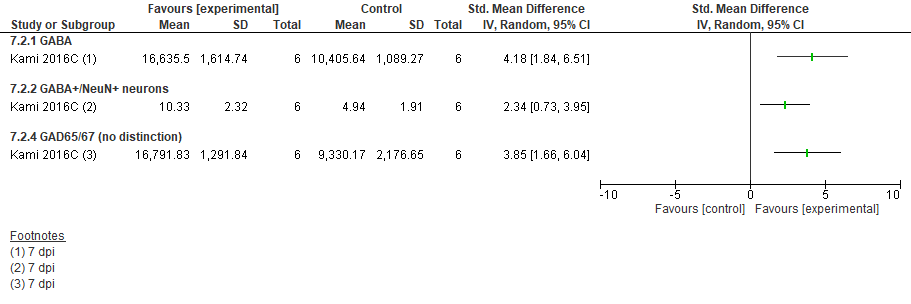**  **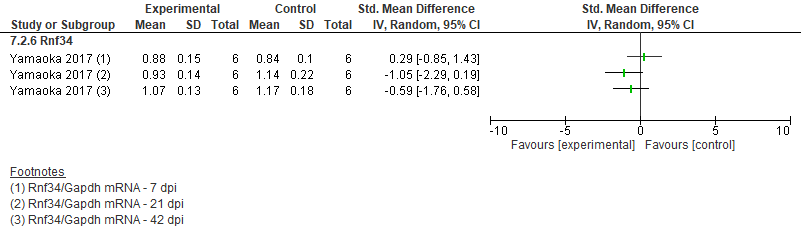** |
| **C8. Dopamine** |
| **BRAINSTEM** |
| **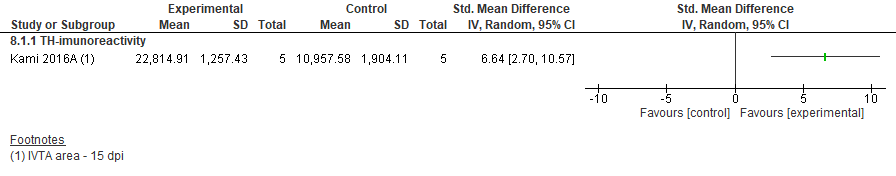** |
| **C9. Purine** |
| **SPINAL CORD (unspecified)** |
| **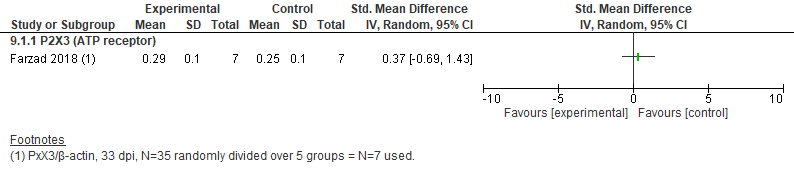** |
| **C10. Opioid system** |
| **BRAINSTEM** |
| **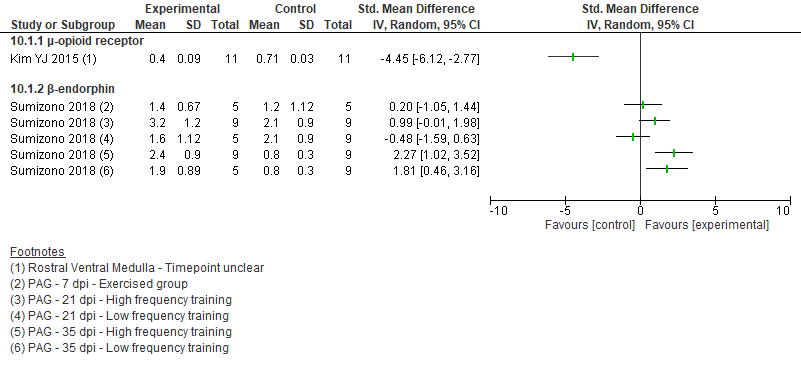** |
| **SPINAL CORD (unspecified)** |
| **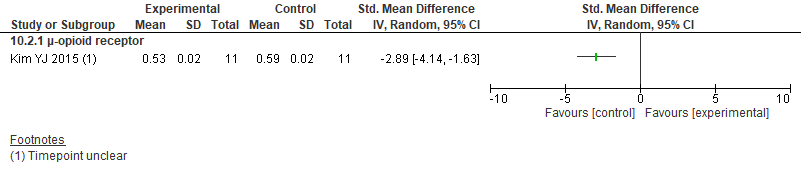** |
| **DORSAL HORN** |
| **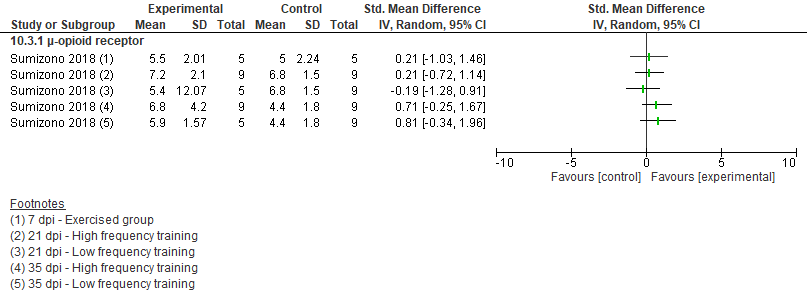** |
| **C11. NEUROPEPTIDES** |
| **DORSAL HORN** |
| **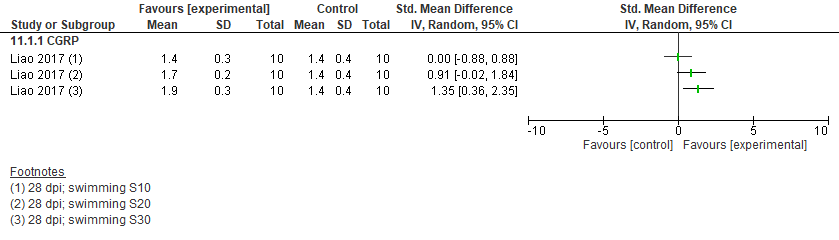**  **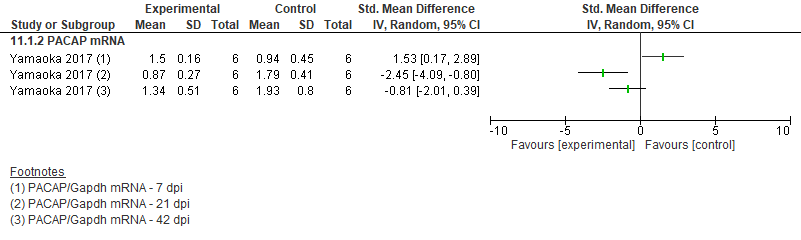** |
| **BONE** |
| **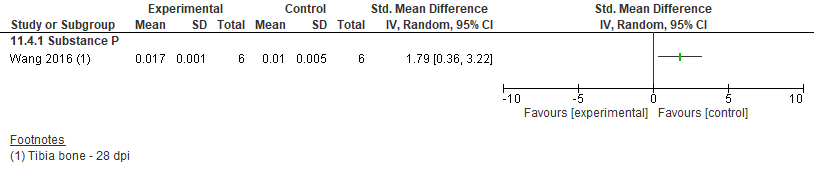** |

| **(SUB)EPIDERMIS** |  |
| --- | --- |
| **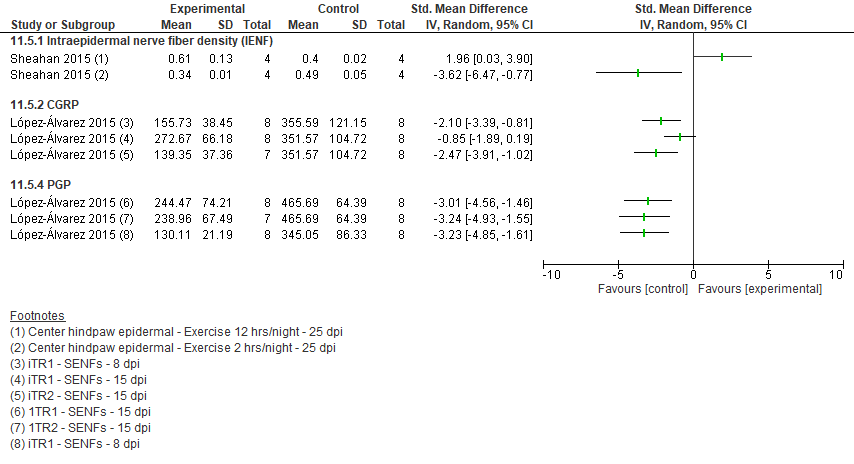** |  |
| **C12. SYNAPTIC STRIPPING** |  |
| **DORSAL HORN** |  |
| **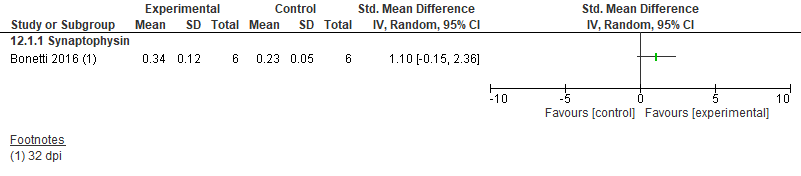** |  |
| **VENTRAL HORN (Motoneurons)** |  |
| **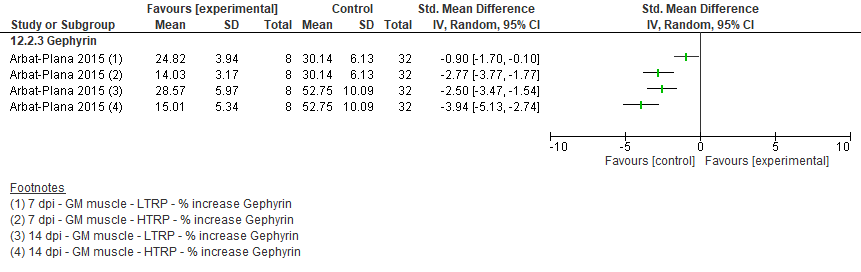**  **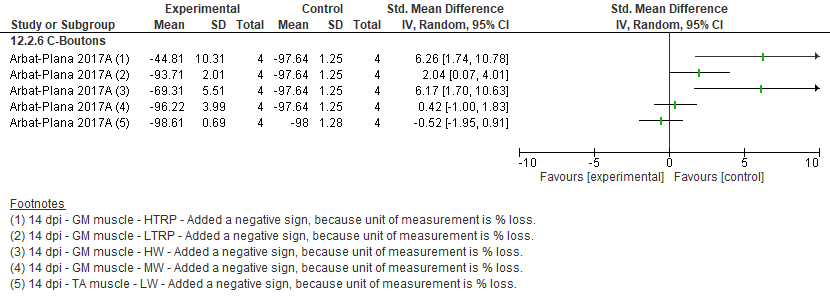** |  |
| **OTHER** |  |
| **C13. Potassium-Chloride Cotransporters (KCC)** | |
| **DORSAL HORN** | |
| 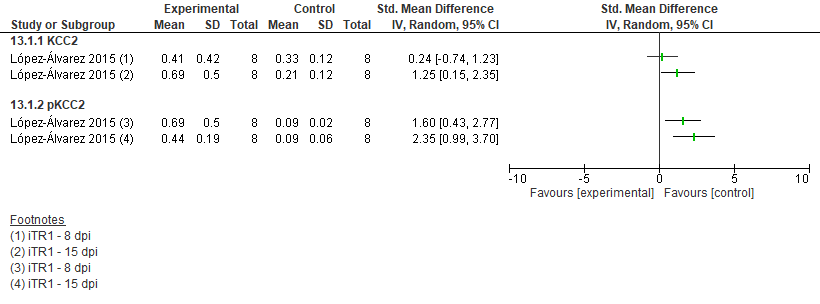 | |
| **Dorsal Root Ganglion** | |
| 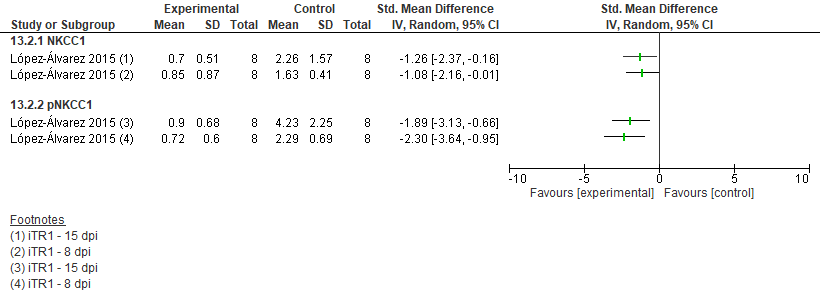 | |
| **C14. MAPK signaling pathway** | |
| **NERVE** | |
| 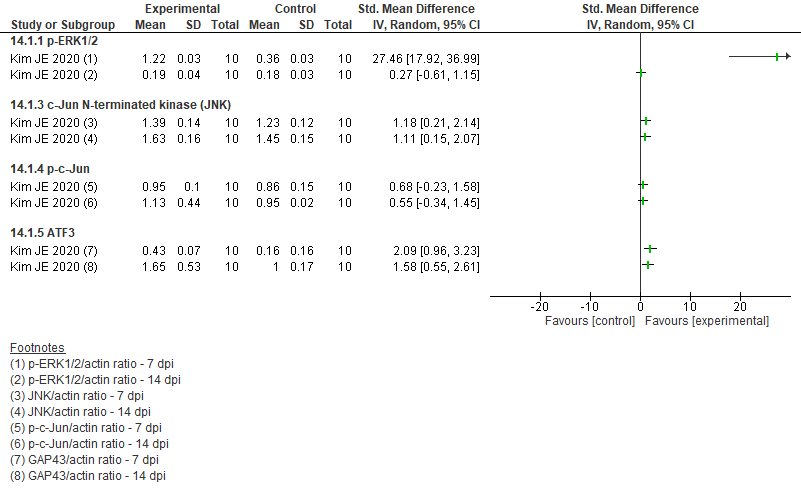  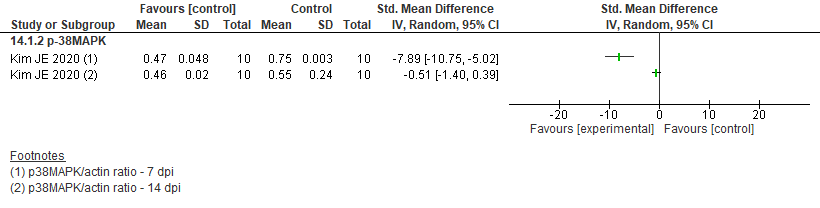 | |
| **C15. GAP43** | |
| **Dorsal Root Ganglion** | |
| 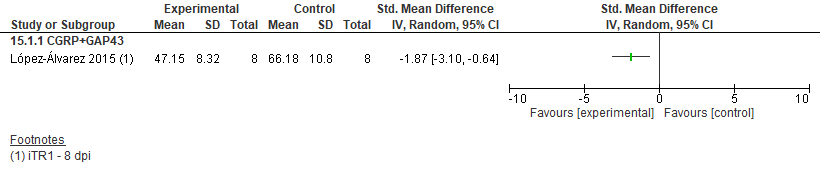 | |

| **(SUB)EPIDERMIS** |
| --- |
| **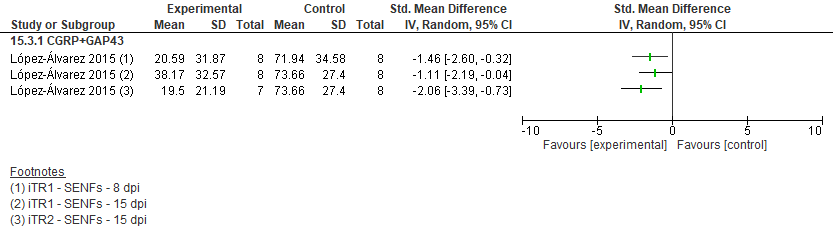** |
| **C16. CREB** |
| **BRAINSTEM** |
| 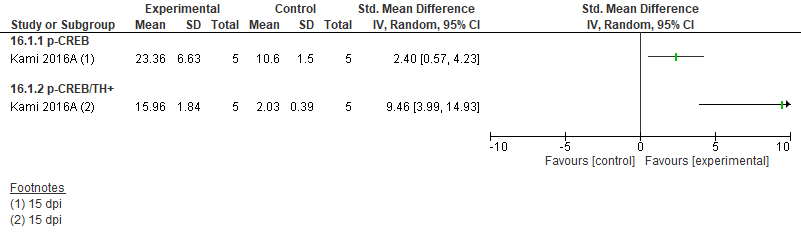 |
| **DORSAL HORN** |
| 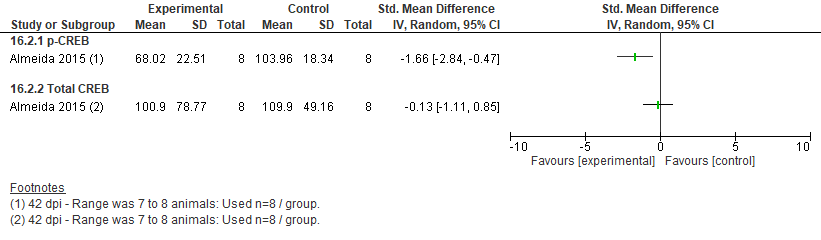 |

| **NERVE** |
| --- |
| 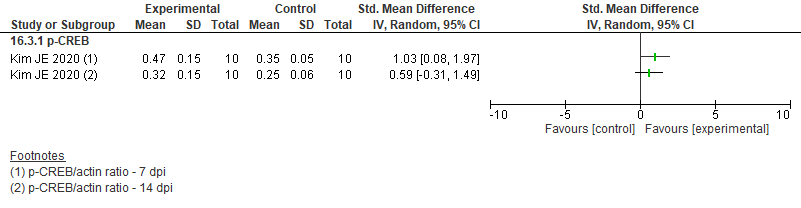 |
| **C17. Oxidative Stress** |
| **SERUM** |
| 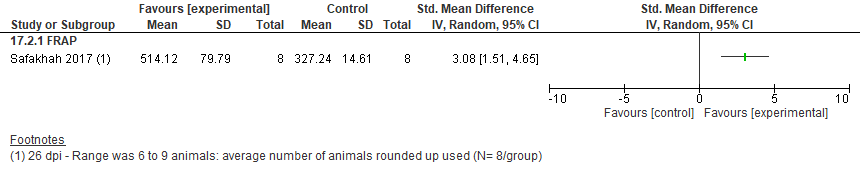  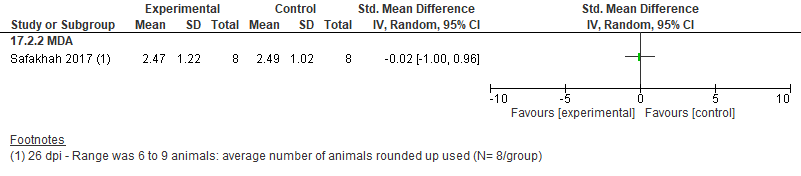 |
| **C18. PLCy-1** |
| 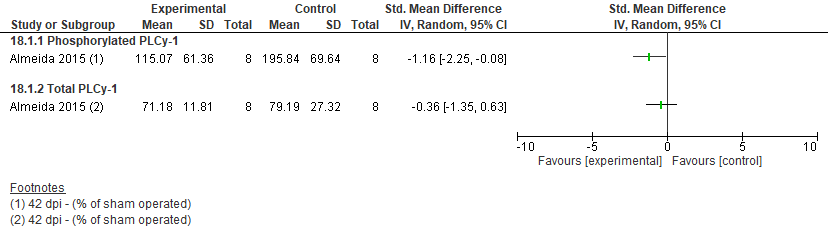 |
